# Poly-specific neoantigen-targeted cancer vaccines delay patient derived tumor growth

**DOI:** 10.1186/s13046-019-1084-4

**Published:** 2019-02-14

**Authors:** Luigi Aurisicchio, Erika Salvatori, Lucia Lione, Silvio Bandini, Matteo Pallocca, Roberta Maggio, Maurizio Fanciulli, Francesca De Nicola, Frauke Goeman, Gennaro Ciliberto, Antonella Conforti, Laura Luberto, Fabio Palombo

**Affiliations:** 1 Takis, Rome, Italy; 2 Biogem, Ariano Irpino, Italy; 3 Università Magna Grecia, Catanzaro, Italy; 4 UOSD SAFU, IRCSS Regina Elena National Cancer Institute, Rome, Italy; 5 Oncogenomic and Epigenetic Unit, IRCCS - Regina Elena National Cancer Institute, Rome, Italy; 6 IRCCS Regina Elena National Cancer Institute, Rome, Italy; 7 Evvivax, Rome, Italy; 8 Vitares, Rome, Italy

**Keywords:** Immunotherapy, T cells, Affinity, Cancer vaccine, Neoantigen, Vaccination, Electroporation

## Abstract

**Background:**

Personalized cancer vaccines based on neoantigens have reached the clinical trial stage in melanoma. Different vaccination protocols showed efficacy in preclinical models without a clear indication of the quality and the number of neoantigens required for an effective cancer vaccine.

**Methods:**

In an effort to develop potent and efficacious neoantigen-based vaccines, we have developed different neoantigen minigene (NAM) vaccine vectors to determine the rules for a successful neoantigen cancer vaccine (NCV) delivered by plasmid DNA and electroporation. Immune responses were analyzed at the level of single neoantigen by flow cytometry and correlated with tumor growth. Adoptive T cell transfer, from HLA-2.1.1 mice, was used to demonstrate the efficacy of the NCV pipeline against human-derived tumors.

**Results:**

In agreement with previous bodies of evidence, immunogenicity was driven by predicted affinity. A strong poly-functional and poly-specific immune response was observed with high affinity neoantigens. However, only a high poly-specific vaccine vector was able to completely protect mice from subsequent tumor challenge. More importantly, this pipeline - from the selection of neoantigens to vaccine design - applied to a new model of patient derived tumor xenograft resulted in therapeutic treatment.

**Conclusions:**

These results suggest a feasible strategy for a neoantigen cancer vaccine that is simple and applicable for clinical developments.

**Electronic supplementary material:**

The online version of this article (10.1186/s13046-019-1084-4) contains supplementary material, which is available to authorized users.

## Background

Cancer immunotherapy based on immune checkpoint inhibitors (ICI) proved to be significantly successful in the treatment of tumors with poor prognosis [1]. Antibodies targeting the PD1/PDL-1 or CTLA-4 pathways are likely to act by rescuing cytotoxic T cell responses against mutation-derived antigens, known as neoantigens [2]. However, the immune responses induced by ICI are suboptimal as indicated by the wider immune repertoire detected by priming PBMCs from healthy individuals with cancer-specific neoantigen peptides [3]. Recent evidence in cancer patients has shown that the T cell repertoire of immunogenic neoantigens induced by neoantigen cancer vaccines (NCV) only partially overlaps the specificity reactivated by ICI [4, 5]. Therefore, treatment with ICI does not release all the potential cancer-specific immune responses, leaving room for new therapeutic approaches.

Preclinical studies highlighted the feasibility of targeting mutation-derived neoantigens by a personalized cancer vaccine (reviewed in [2]). The current strategy used to target neoantigen cancer vaccine (NCV) was initially reported in the B16 melanoma model where the possibility of inducing an effective immune response targeting neoantigens by a cancer vaccine was shown [6] .

Naked DNA delivered in combination with electroporation (DNA-EP) is considered an efficient delivery system [7] that has moved from preclinical to clinical settings in cancer vaccines as well as in viral vaccine applications (reviewed in [8]). Even though there are 10 ongoing clinical trials registered with www.clinicaltrial.gov [1, 2, 8] using this technology, there have been no studies that have characterized the DNA-EP delivery of minigenes encoding a string of neoantigens in preclinical tumor models so far. In contrast, many reports in preclinical models support the efficacy of other vaccine methods based on peptides [6] or RNA [9, 10].

Knowing how to predict immunogenicity of neoantigens is still an ongoing debate. The difference between predicted binding affinity to MHC of mutated epitope vs. the natural epitope has been proposed as a relevant factor [11]. This concept was initially explored with peptide vaccines in sarcoma and fibrosarcoma tumor models. The rationale underlying this notion is that the immune response induced by CD8 cells against neoantigens could have been eliminated by immunological tolerance at the central and/or periphery level against the corresponding wild-type (WT) epitope. The author defined this parameter as a differential agretopic index (DAI). The quality of neoantigens has also been explored from a different perspective. In an attempt to establish correlations between immune responses and different subclasses of neoantigens, it has been proposed that there may be similarities with viral epitopes that can favor better immune responses [12]. Interestingly, this initial evidence was further supported by clinical studies where correlations were established between long term survival pancreatic cancer patients and immune responses against viral-like neoantigens [13]. In general, these sets of evidence underlie the need for a better understanding of vaccine induced immune responses against neoantigens. Here, we investigated how the quality and the number of neoantigens affects immunogenicity and anti-tumor activity of neoantigen minigene (NAM) vaccines delivered by DNA-EP in murine tumor models and further showed that this approach is effective in patient derived tumors.

## Methods

### Cell lines and mice

The B16 melanoma and MC38 colon carcinoma cell lines were purchased from ATCC. Master and working cell banks were generated upon receipt, of which the third and fourth passages were used for all tumor challenge experiments. Cells were mycoplasma free as per internal regular controls. Transfection was carried out with Lipofectamine 2000 according to the manufacturer’s instructions.

6–8 week old C57BL/6 female mice or Rag2^−/−^ Il2r ^−/−^ mice (Envigo) were housed in the Plaisant animal house according to the national legislation and kept in standard conditions in accordance with Takis’s ethics committee approval. HHK mice expresses the α1 and α2 domain of human HLA-A0201 fused to the α3 domain of H-2K^b^ and were generated in our laboratory (manuscript in preparation).

### Genomic procedure for neoantigen sequencing

Neoantigen sequences were selected from available data for the MC38 [14] and B16 cells [6, 9]. Sequences of selected neoantigens were confirmed by RNAseq analysis for the MC38, U11 and M285 cells and by NGS target resequencing for B16 cells. For the human derived tumor models, neoantigens were selected according to expression data from RNA sequencing, which was performed as described earlier [15]. In short, total RNA was extracted from tumor cells in culture or from a tumor of 100 mm^3^ implanted s.c., ribosome depleted by Ribo Zero Gold and prepared for sequencing using the TruSeq Stranded Total RNA Sample Prep kit (Illumina, Inc., San Diego, CA, USA) following the manufacturer’s instructions. The quality of the libraries obtained was monitored through using a Bioanalyzer, and quantity by qPCR. Sequencing in paired-end mode (2 × 76) was performed on a NextSeq500 (Illumina, Inc., San Diego, CA, USA). The genomic regions encompassing the mutations reported previously for the B16 cell line [6, 9] were amplified by PCR with the primers indicated in Additional file 1: Table S1, controlled on a gel for their specificity and quantity. Subsequently, the PCR products were pooled and purified (QIAquick PCR purification kit, Qiagen, Valencia, CA, USA). 10 ng of the amplicons were further processed using the TruSeq ChIP Library Preparation kit (Illumina, Inc., San Diego, CA, USA) and sequenced on a NextSeq500 (Illumina, Inc., San Diego, CA, USA).

### Bioinformatic procedure for neoantigen selection and prioritization

The RNA-Seq reads were processed with the cloud pipeline RAP [16] in order to assess quality measures, and map reads to the mouse genome (vv. mm9). Subsequently, we performed variant calling with Freebayes [17] (default parameters). The coverage of each locus of interest was extracted from the resulting VCFs. RPM were calculated by normalizing both read depths with the amount of mapped reads for each library (RPM = (coverage*1e6)/(total_mapped_reads)). For U11 and M285 primary human tumors, we processed RNA-seq reads with the same pipeline, obtaining 45,749,750 and 24,083,207 total mapped reads, that we subsequently analyzed for variants. The mapped reads were, for in vitro and in vivo MC38 samples, respectively 29,610,045 and 34,407,355. The expressed epitopes were calculated from the expressed mutation list with our in-house pipeline Narciso. MHC binding affinity was extracted via Net-MHC4 [18] and the DAI was calculated as a ratio of predicted binding affinity of wild type amino acid sequence and the cognate neoantigen.

### Vaccine and mouse models

DNA vaccines were generated using codon optimized DNA minigenes encoding 9 or 27 amino acids as it is listed in Table 1, Table 2, Additional file 1: Tables S2, S4 and S5. In the 27 mer epitope minigenes, the mutated amino acid was in a central position. The peptide sequence was back-translated according to the mouse optimized codon usage and linked to amino acid spacers, i.e. REKR, recognized by the furin protease as previously described [7]. Synthetic genes and expression vectors were generated at Eurofins using pTK1 as a backbone vector, which drives the expression of poly-specific neoantigen expression cassette under the human CMV promoter and enhancer. Control vaccine vectors are the empty pTK1 vectors or pTK1-CEAs, which express the codon optimized sequence for the full length CEA protein as described previously [19]. DNA-EP was carried out as previously described [20]. Peptide vaccination was performed by subcutaneous injection of a mixture of 100 μg peptide and 50 μg CpG-ODN (Sigma) in Incomplete Freund’s Adjuvant (IFA) per mouse. Tumor challenge of mouse models was performed by injecting 3 × 10^5^ MC38 cells or 2 × 10^5^ B16 cells s.c. in the right flank of the mice.

**Table 1:** M1 vaccine expressing MC38 neoantigens (see Fig. 2)

		WT Peptide			Neoantigen				
#	Symbol	Seq	H2-Db	H2-Kb	Seq	H2-Db	H2-Kb	DAI	Imm.Resp.
1	Wbp7	SNFHFMCAR	39,828	499	SNFHFMCA**L**	7213	**10**	**> 5**	+
2	Hace1	QINAFLQGF	44,767	1128	QI**Y**AFLQGF	37,598	**39**	**> 5**	+
3	Hdgfrp2	KGYPHWPAR	43,395	4131	KGYPHWPA**L**	12,706	33	**> 5**	–
4	Kpna6	CTLQFEAAW	34,408	1319	CTLQFEAA**L**	8030	42	**> 5**	–
5	Aurkaip1	RTRFLRRKV	45,504	6329	RTRFLR**L**KV	42,713	7062	1	–
6	Srebf2	HSFVDSVGF	33,887	3985	HSFV**Y**SVGF	38,481	135	**> 5**	–
7	Mttp	TGYVERSPR	41,635	11,661	TGYVERSP**L**	9548	131	**> 5**	–
8	Nle1	MALSTDYAL	1982	1278	MALST**Y**YAL	407	46	**> 5**	+/−
9	Zbtb24	SLLEHMSLH	43,119	18,788	SLLEHMSL**L**	7649	265	**> 5**	–
10	Hnrnpf	GYVVKLRGL	42,720	3065	**S**YVVKLRGL	38,966	438	**> 5**	–

Predicted binding are expressed as nM values according to NetMHC-4. Immunogenic neoantigens (+) and intermediate response (+/) are indicated. Bold residues in the peptide sequence are mutated

**Table 2:** M2 vaccine expressing MC38 neoantigens (see Fig. 3)

		WT Peptide			Neoantigen				
#	Symbol	Seq	H2-Db	H2-Kb	Seq	H2-Db	H2-Kb	DAI	Imm. Resp.
11	Tmem135	FALMNRKAL	12	1426	FALMN**L**KAL	**12**	681	< 5	+
12	Aatf	MAPIDHTAM	297	5239	MAPIDHT**T**M	90	6675	> 5	–
13	Spire1	SAIRSYQDV	814	271	SAIRSYQ**Y**V	**28**	**43**	**> 5**	+
14	Zbtb40	KSFHFYCRL	7827	4	KSFHFYC**P**L	1318	4	< 5	–
15	Slc12a4	LSAARYALL	26,865	16	LSA**S**RYALL	27,697	14	< 5	–
16	Nfe2l2	ASYSQVAHI	4627	66	ASY**S**LVAHI	1309	23	< 5	–
17	Herc6	CGYEHTAVL	7636	140	C**V**YEHTAVL	9017	91	< 5	–
18	Copb2	MSYFLQGKL	6858	72	MSYFLQG**T**L	2039	51	< 5	–
19	Reps1	AQLPNDVVL	89	9261	AQL**A**NDVVL	**16**	8591	**> 5**	+
20	Adpgk	ASMTNRELM	6	2267	ASMTN**M**ELM	**4**	1288	< 5	+

Predicted binding affinity by NetMHC-4 software. Immunogenic neoantigens (+) are indicated. Mutated residues are in bold

To test our approach with human primary cancers a new model was established. Immunocompetent mice transgenic for HLA-A0201 (HHK) were vaccinated with neoantigens encoding DNA vaccines and 20 × 10^6^ splenocytes transferred in the peritoneum of immunodeficient Rag2^−/−^ Il2r−/− recipient mice bearing human derived tumors. Screening for the expression of the HLA-2.1 resulted in the selection of the U11 lung cancer tumor model [21] and M285 melanoma tumor model [22],which are low passage human cell lines. For tumor growth 5 × 10^6^ cells were injected s.c. and followed over time. All national and institutional guidelines were followed and experiments were approved by governmental authorities (authorization n. 292/2016/PR). All mouse experiments were repeated at least twice with a variable number of animals as described in the figure legends.

### Immune responses

T-cell peptides-specific poly-functionality responses were determined by using intracellular cytokine staining (ICS) performed by flow cytometric detection. Briefly, PBMCs or splenocytes harvested from immunized mice (or controls) were incubated for 10 min at room temperature in ACK (Ammonium-Chloride- Potassium) Lysing Buffer (Life Technologies) and then washed in RPMI-1640 medium (Gibco-BRL) with 10% fetal bovine serum (FBS). Blood was retro-orbital retrieved in a volume of 100,200 ul and processed, at least 1 × 10^6^ PBMCs or splenocytes were cultured in 96-well plates and stimulated for 12–16 h in 10% FBS-supplemented RPMI-1640 medium containing 1 μg/ml of Brefeldin A (Sigma-Aldrich, St Louis, MO, USA), and 10 μg/ml of the single peptides or the indicated pool of peptides in a 1:1 cells/peptide ratio at 37 °C. Following stimulation and surface staining, samples were then fixed and permeabilized by using the Cytofix/Cytoperm kit (BD Biosciences, San Jose, CA, USA). We excluded dead cells by using the Violet Dead cell stain kit (Invitrogen, Carlsbad, CA, USA). PBMC or splenocytes were incubated with anti-Fcγ receptor (2.4G2) followed by surface staining with anti-CD3e (142-2C11), anti-CD4 (RM4–5) and anti-CD8 (53–6.7,) all antibodies were purchased from BD Biosciences (San Jose, CA, USA). Subsequently, the cells were intracellularly stained with the following antibodies: anti-IFNγ (XMG1.2), anti-IL-2 (JES6-5H4) and anti-TNFα (MP6-XT22; all from eBioscience, San Diego, CA, USA). The stained samples were acquired through a CytoFLEX flow cytometer (Beckman Coulter), and the data were analyzed using CytExpert software (version 2.1) with the gating strategy reported in Additional file 2: Figure S1. Effector memory T cells were evaluated as CD44^+^CD62L^low^ using the anti-CD44 (IM7) and anti CD62L (MEL14) from eBioscience, San Diego, CA, USA and gated on CD3^+^CD8^+^IFNγ^+^ T cells. Gating strategy is depicted in Additional file 2: Figure S1.

### IFN-γ ELIspot

The assay was performed according to the manufacturer’s instructions (U-Cytech, Utrecht, Netherlands). Briefly, standard 96-well plates (Millipore) were coated with anti-mouse IFNγ antibody diluted 1:200 in sterile PBS (final conc. 10 μg/ml). Splenocytes were plated at 4 × 10^5^ and 2 × 10^5^ cells/well, in duplicate, with MC38 neoantigens, Reps1, both WT and mutated at decreasing concentration from 1 pM to 100 μM. After overnight stimulation at 37 °C, plates were washed and incubated with biotinylated anti-mouse IFNγ antibody, washed and incubated for 2 h at room temperature with streptavidin-AP conjugated antibody. After extensive washing, 50 μl/well of the substrate (NBT/BCIP-1 step solution, Pierce) was added to measure spot development. The washing plates were thoroughly washed with distilled water to stop the reaction. Plates were allowed to air-dry completely, and spots were counted using an automated ELISPOT reader (Aelvis ELIspot reader, A.EL.VIS Gmbh, Germany).

### Statistical analysis

Log-rank test, ANOVA and two-tailed Student’s t-tests were utilized where indicated. All analyses were performed in JMP version 5.0.1 (SAS Institute, Cary, NC).

## Results

### High affinity drives immunogenicity of NCV delivered by DNA-EP

**Fig. 1:**
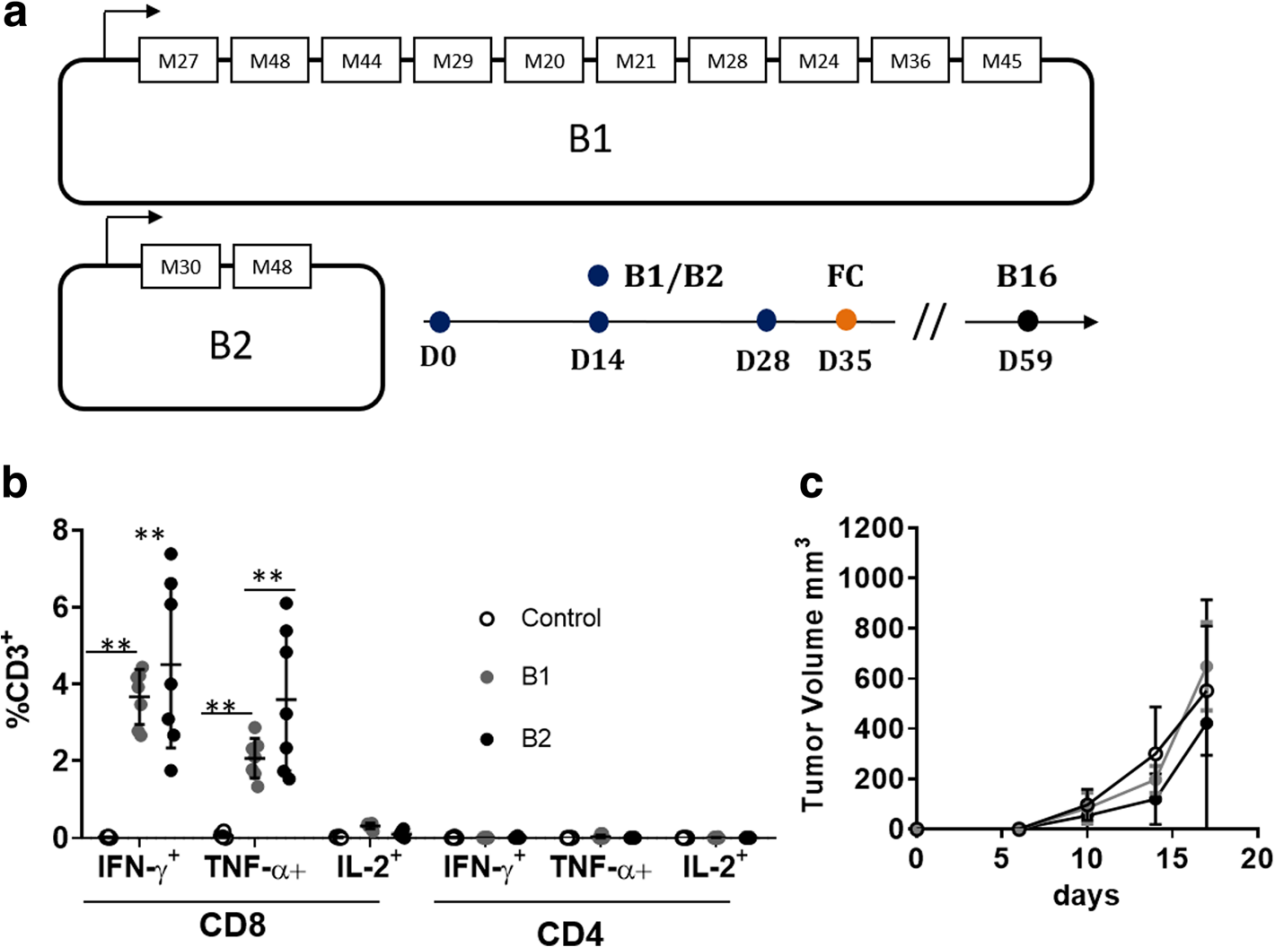
DNA vaccine delivered by EP induces immune responses against neoantigens of B16 tumor model. Six mice per group were vaccinated with three biweekly injections of the B1 or the B2 vaccine (V), which encode neoantigens from the B16 cells in the form of 27 mer peptide as reported in Additional 1: Table S2. (**a**) Scheme of B1 and B2 vaccine vectors, which encode for ten and two neoantigens, respectively, numbers correspond to neoantigens listed in Additional file 1: Table S2. (**b**) One week after last immunization, T cell immune responses were analyzed by FC in the peripheral blood for the expression of IFN-γ and TNF-α by FC, gating strategy is shown in Additional file 2: Figure S1, **p < 0.001 Mann-Whitney test. (**c**) On day 59 six mice per group were challenged with B16 cells (B16) and tumor growth followed over time as described in M&M, bars represent SD

To develop a pipeline process for NCV based on DNA-EP, we first asked whether published neoantigens previously delivered in the form of a peptide or an RNA were effective through using our technology platform. Starting from data in the literature [9, 23], we generated NAM expressing neoantigens from the B16 melanoma cell line (Fig. 1). The B1 vector expresses 10 neoantigens while the B2 expresses only two neoantigens, M30 and M48, with the last one also expressed in B1. We were able to detect immune responses against the pool of neoantigens in the peripheral blood (Fig. 1b) and at the level of a single neoantigen in the splenocytes by flow cytometry (FC) against two out of the eleven neoantigens (Additional file 1: Table S2). The immune response against the M48 neoantigen was similar in mice vaccinated with B1 or B2 vaccine vectors suggesting that the presence of additional neoantigens in The B1 vaccine vector does not affect immunogenicity (data not shown). Vaccinated mice were not protected from tumor challenge (Fig. 1c) while mutations were confirmed to be present by genomic sequencing (see M&M). We noticed that the predicted binding values for ten out of eleven neoantigens were over 500 nM (Additional file 1: Table S2) and a recent pan-cancer analysis suggests that immunogenicity of neoantigens is driven by lower predicted binding values [24]. Therefore, we looked at the MC38 tumor model for which high affinity neoantigens have been described [14].

**Fig. 2:**
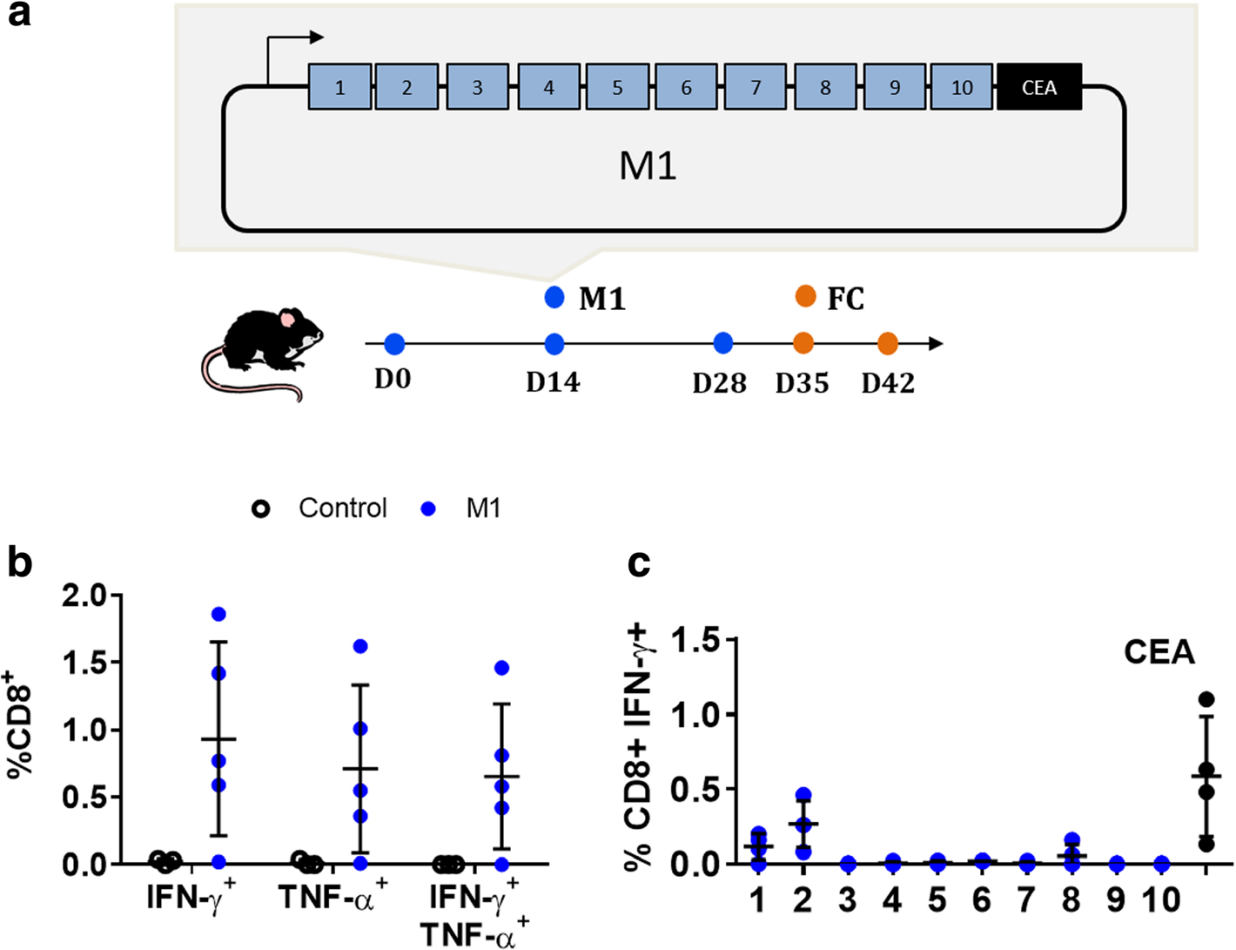
M1 vaccine induces neoantigen specific immune responses against MC38 cells. Mice were vaccinated with three biweekly injections of M1 vaccine, which encodes 10 neoantigens from the MC38 colon cancer and a reference CD8 epitope from CEA, immune responses were evaluated in the periphery blood and in the splenocytes by FC. (**a**) Scheme of M1 vaccine and experimental set up. Numbers in M1 vaccine correspond to neoantigens listed in Table 1. (**b**) One week after last immunization T cell immune responses were analyzed in the peripheral blood for the expression of IFN-γ and TNF-α by FC PBMC were restimulated overnight with a pool of 10 neoantigen peptides from 1 to 10, dots represent value of single mice the gating strategy is shown in Additional file 2: Figure S1. (**c**) On day 42 immune responses against single peptides were analyzed by FC in restimulated splenocytes of four mice for the expression of IFN-γ, dots represent value of single mice

To explore the impact that the quality of neoantigens has on immunogenicity, we looked at two classes of neoantigens delivered in the context of NAMs by DNA-EP vaccination, [7] neoantigens with predicted high or low affinity according to a threshold of 50 nM [24]. To this end, we generated two vaccine vectors, M1 and M2, encoding twenty neoantigens from MC38 colon cancer cells [14]. Neoantigen expression in our in vitro and in vivo samples was confirmed by the RNAseq analysis (Additional file 1: Table S3). Nine neoantigens encoded in the M1 vaccine (Fig. 2a) have a predicted affinity at least five-fold higher than that of the corresponding WT peptide (ratio WT/mut > 5), indicated as DAI (Table 1).

Mice were vaccinated with three biweekly vaccinations and immune responses were analyzed by the FC analysis in the peripheral blood on day seven after the last treatment. Significant immune responses through CD8^+^IFN-γ^+^, CD8^+^TNFα^+^ and poly-functional CD8^+^IFN-γ^+^TNFα^+^, were observed by FC against the pool of neoantigen peptides in the peripheral blood (Fig. 2b). Mice were then sacrificed on day forty-two and immune responses analyzed at the level of a single neoantigen in restimulated splenocytes (Fig. 2c). CD8^+^IFN-γ^+^ T cells were detected against two neoantigens, Wbp7 and Hace1. Of note, two neoantigens out of the five with predicted high affinity (< 50 nM) but none of the five neoantigens with predicted low affinity (> 50 nM) were immunogenic. To exclude that the result could be influenced by the position of neoantigens and the length of DNA construct, we included a CD8 epitope reference from the Carcino-Embryonic Antigen (CEA) at the end of the open reading frame. Frequency of CEA specific CD8 immune responses induced by the minigene construct was similar to the full-length CEA protein (Additional file 2: Figure S2) [19]. These data validate the architecture with ten neoantigens and moreover suggest that high affinity is the driving force behind immunogenicity.

**Fig. 3:**
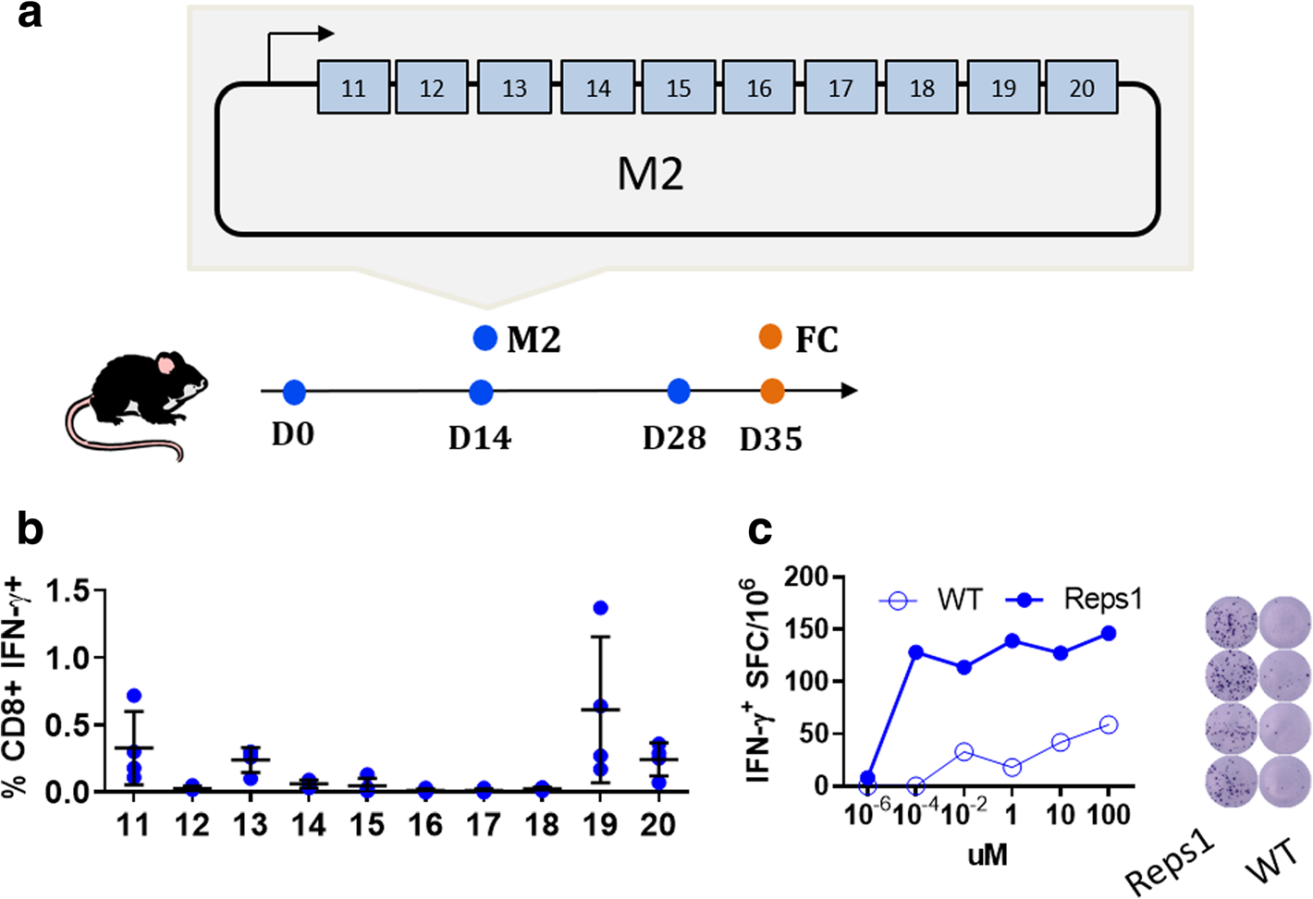
High affinity MC38 neoantigens are immunogenic. Mice were vaccinated as reported in the scheme with the M2 vaccine, which encodes for the high affinity neoantigens listed in Table 2. (**a**) Scheme of M2 vaccine. (**b**) One week after the last vaccination four mice were sacrificed and M2-specific immune responses were analyzed in the splenocytes, value refers to % of CD8^+^IFN-γ^+^ gated on CD3^+^ T cells and measured by FC with the neoantigens peptides listed in Table 2. (**c**) IFN-γ ELISPOT analysis with splenocytes restimulated with increasing concentration of Reps1 neoantigen and cognate WT peptide the graph on the left shows the number of IFN-γ producing cells after in vitro stimulation of 4 × 10^5^ splenocytes with increasing concentration of peptide; image on the right shows quadruplicate results at 10^− 4^ μM peptide concentration

To further support the immunogenicity of high affinity neoantigens, we generated the M2 vaccine (Fig. 3a). Table 2 shows that seven of the neoantigens encoded by the M2 vaccine are characterized by high affinity (Tmem135, Aatf, Spire1, Reps1, Adpgk Zbtb40, Slc12a4, Nfe2l2), and three with a value higher than 50 nM (Aatf, Herc6, Copb2). Table 2 reported also on the DAI to compare the previous vector and to verify whether the neoantigens with high affinity and DAI are more immunogenic.

Splenocytes from vaccinated mice showed a CD8^+^ IFN-γ^+^ specific T cell response against four out of seven high affinity neoantigens: Tmem135, Spire1, Reps1 and Adpgk (Fig. 3b). The cumulative data obtained with B1, B2, M1 and M2 vaccine vectors delivered by NAMs via DNA-EP demonstrate that higher frequency of immunogenic neoantigens are observed in the presence of predicted high affinity (6/12) with respect to predicted lower affinity (2/19) (*p* < 0.05 one tailed Mann-Whitney test). Among the immunogenic neoantigens there is a trend favoring those with positive DAI (4/6 have a DAI > 5). The limited number of immunogenic neoantigens tested prevents us to come to any conclusions on the impact of DAI on immunogenicity of neoantigens delivered by DNA-EP. To prove the specificity of DNA-EP delivered neoantigens, we compared the immune responses of a neoantigen with that of the cognate epitope. The IFN-γ ELISPOT analysis for the Reps1 neoantigen showed a clear specificity for the neoantigen compared to the WT peptide (Fig. 3c). The difference was more evident in restimulated splenocytes with a decreasing concentration of peptides. Similar results were observed in the peripheral blood as measured by the FC (Additional file 2: Figure S3).

**Fig. 4:**
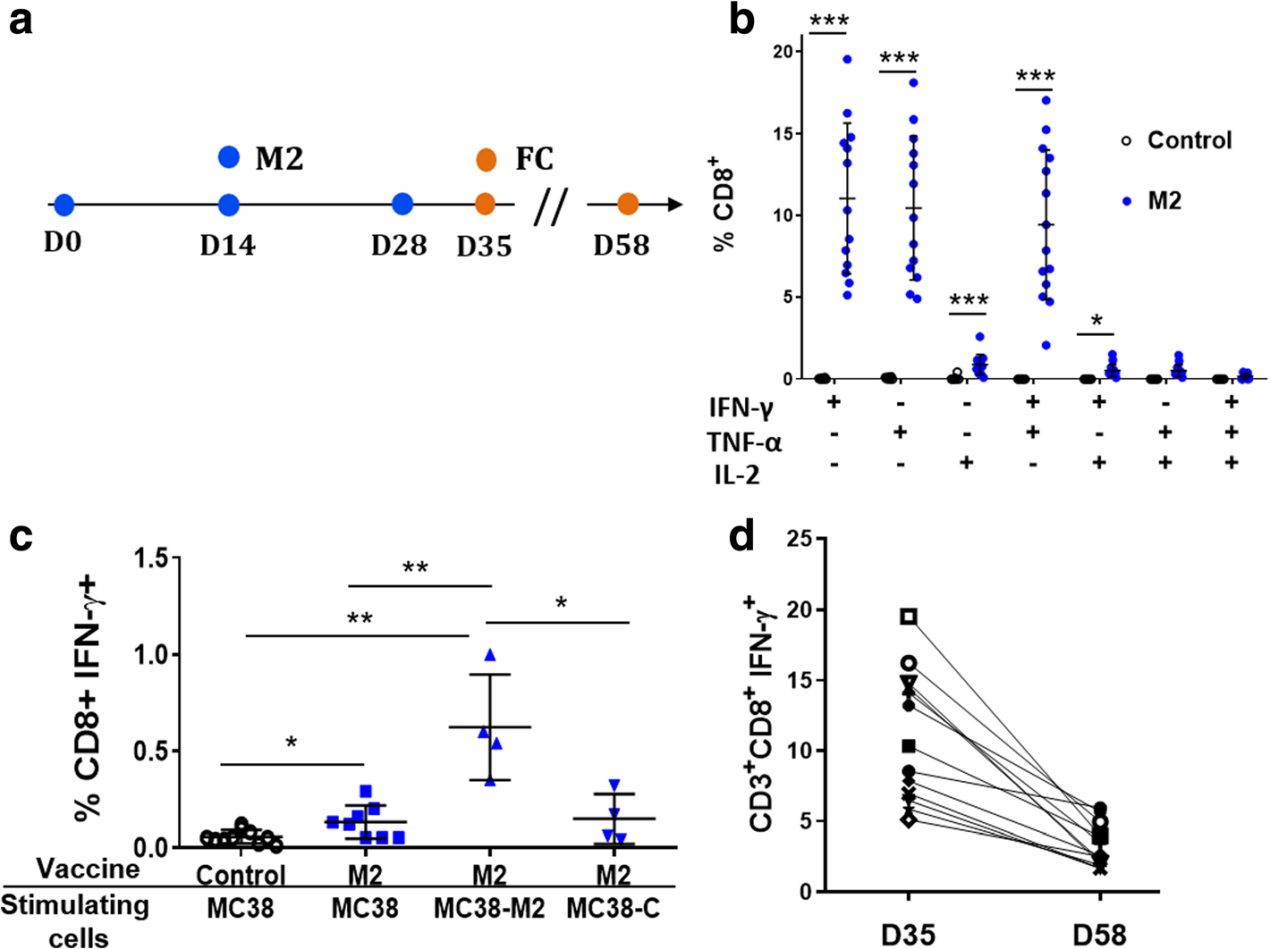
M2 vaccine elicits a poly-functional and long lasting immune response. (**a**) Experimental set up: mice were vaccinated with the M2 vaccine by DNA-EP once every two weeks for three times and immune responses were analyzed on day 35 and 58. (**b**) The immune response was measured in the peripheral blood by FC on day 7 after the last vaccination. Paired matched T-test 2-tailed *p < 0.05, **p < 0.01, ***p < 0,001, ****p < 0,0001. (**c**) Splenocytes from the M2 vaccinated mice are activated by MC38 cells. Mice were vaccinated according to the scheme and splenocytes were collected on day 35. Splenocytes from mice treated with a different vaccine control (pTK1) or M2 (upper row) were incubated with diverse stimulating cells (lower row) MC38 cells (MC38), with MC38 cells transfected with the M2 vaccine (MC38-M2) or MC38 cells transfected with an unrelated vaccine (MC38-C). The first group on the left represent the background signal that is given by splenocytes from mice vaccinated with control plasmid and stimulated with MC38 (Control/MC38). Dots represent IFN-γ production of splenocytes from single mice measured by FC, median and SD, *p < 0.05, **p < 0.01 Mann-Whitney test. (**d**)The analysis of immune responses was performed on day 7 (D35) and on day 30 (D58) after the last vaccination in the peripheral blood by ICS. Dots represent values of individual mice of two independent experiments with six-seven mice per group

We then asked whether the CD8+ T cells induced by DNA-EP against the MC38 specific neoantigens could recognize cancer cells. To do this, mice were vaccinated with the M2 vaccine vector as described in Fig. 4a and the FC analysis was performed on day 7 after the last vaccination. A strong immune response against the M2 peptide pools was observed via poly-functional CD8^+^IFN-γ^+^TNFα^+^, CD8^+^TNFα^+^IL2^+^ and CD8^+^IFN-γ^+^TNFα^+^IL2^+^ T cells (Fig. 4b). In order to verify whether the M2 neoantigens were naturally processed and presented, splenocytes from vaccinated mice were incubated overnight with MC38 cells. The comparison between the response induced in control mice to animals vaccinated with M2 vector showed a statistically significant increase in CD8^+^ IFN-γ^+^ T cells upon incubation with MC38 cells, suggesting that M2 neoantigens are present on the cell surface and are specifically recognized by M2 vaccinated mice. (Fig. 4c). Moreover, the frequency of CD8^+^ IFN-γ^+^ T cells further increased when splenocytes were incubated with MC38 cells transfected with the M2 vaccine as compared to untransfected MC38 cells or MC38 cells transfected with a control plasmid. We cannot exclude that mice vaccinated with the M2 vaccine can develop immune responses against additional cryptic epitopes, which could be present when cells are transfected with the M2 plasmid. However, higher immune responses detected with the M2 transfected MC38 cells support the concept that the expression level of a neoantigen is an important aspect of tumor recognition. As expected the percentage of neoantigen-specific CD8^+^IFN-γ^+^ T cells decreased at day 30 but was still measured in the order of single digits (Fig. 4d).

### Poly-functional and poly-specific immune responses protect mice from tumor challenge

**Fig. 5:**
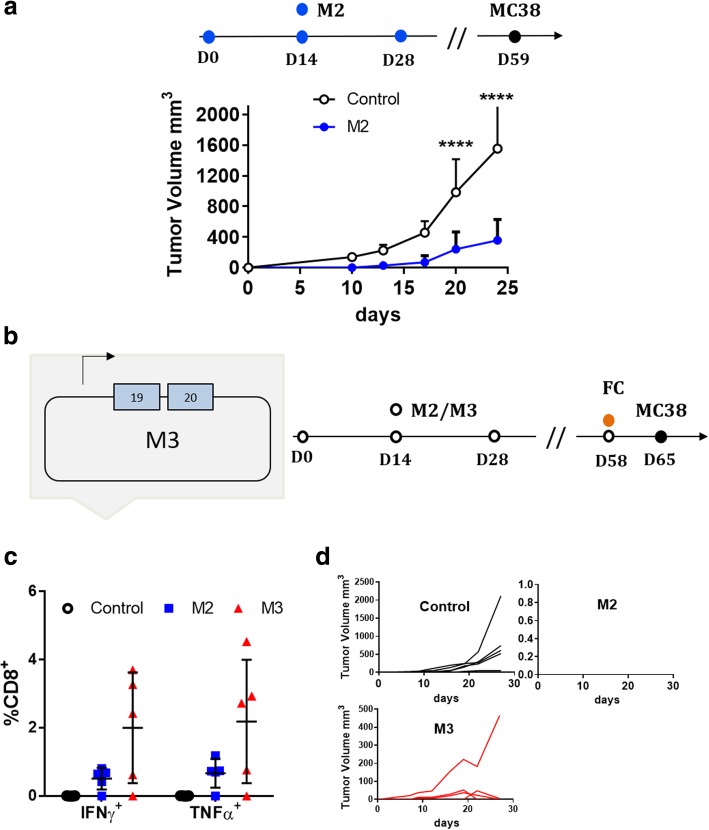
Poly-specificity is key for tumor protection. (**a**) The M2 vaccine delay tumor growth, six mice per group were vaccinated with M2 or left untreated (control) as depicted in the scheme and challenged with MC38 cells on day 59. Tumor growth was significantly reduced in vaccinated mice as compared to the control *p < 0.05 two-way anova bars represent SD. Plots represent value of one out of two experiments. (**b**) To verify the impact of poly-specificity on tumor growth, the M3 vaccine vector was generated for comparing the M2. M3 expresses, the Reps1 and Adpgk neoantigens, which are in common with M2. Regarding the M3 scheme and vaccination protocol, mice were vaccinated either with the M2 or M3 vector at indicated time points and challenged with MC38 cancer cells (MC38). (**c**) CD8^+^ immune responses measured in the peripheral blood by FC on day 58 before vaccination. (D) The representative experiment with five mice per group of the tumor challenge started on day 65, the individual growth curve for MC38 cells is depicted for mice vaccinated with control, M3 and M2 vaccine vectors. The experiments were repeated twice with similar results

To verify whether the use of a poly-functional and poly-specific neoantigen vaccine delivered by DNA-EP platform has an impact on tumor growth, we explored the MC38 tumor model in a prophylactic setting. In fact, the MC38 tumor model is fast-growing and our vaccination protocol with three biweekly DNA-EP is too long to mount a therapeutic immune response (data not shown). For this reason, we focused on tumor prevention rather than on the therapeutic setting. Therefore, on day fifty-nine vaccinated mice were challenged with MC38 cells resulting in a statistically significant delay in tumor growth compared to control mice (Fig. 5a). The analysis of memory T cells at day 59 in an independent experiment revealed that most of the M2 specific T cells were effector memory (CD83^+^CD8^+^IFNγ^+^CD44^+^CD62L^LO^), thus suggesting that a boost in the immune response could further improve tumor protection (Additional file 2: Figure S4). We then asked whether not only an immune response boost but also the degree of poly-specificity would affect tumor growth. To evaluate this aspect, we generated a third vaccine vector, M3 expressing only two immunogenic neoantigens, Dpagt1 and Reps, expressed by the M2 vector and previously identified in MC38 cells by mass spectrometry [14]. We choose these two neoantigens to allow comparison with previous vaccinations, which were carried using peptides. Similar immune responses were observed with Adpgk and Reps1 neoantigens delivered as peptides or as DNA-EP (Additional file 2: Figure S5). Mice were vaccinated as described in Fig. 5b with the M2 or M3 vaccines. To maximize the impact of the vaccine treatment, we performed an immunological boost on day fifty-eight, that is one week before the tumor challenge. Figure 5c shows the immune response at the time of the boost using the peptides as a stimulus for the two neoantigens, Adpgk and Reps1, shared between M2 and M3 vaccine vectors. M3 vaccine induced a slightly higher immune responses, which can be explained by the expression of a lower number of neoantigens. Although the immune responses were not statistically different through CD8^+^IFN-γ^+^ or CD8^+^TNFα^+^ T cells, complete protection from tumor challenge was observed only in mice vaccinated with the M2 vaccine vector (Fig. 5d). These results support the concept that high levels of poly-specificity induced with M2 vaccine via the four immunogenic neoantigens is key in safeguarding mice from tumor take. The efficacy of adjuvant immunotherapy with ICI was recently demonstrated in the clinic for anti PD-1 pembrolizumab [25] and it was previously shown to be effective for the anti CTLA-4 ipilimumab [26]. For comparison purposes we verified whether anti PD1 and anti CTLA-4 could prevent MC38 tumor growth by starting the treatment before tumor challenge. We observed a complete protection from tumor challenge with the anti PD1 and in four out of five animals treated with the anti CTLA-4 antibody (Additional file 2: Figure S6) which is in line with the protection degree of the NCV delivered by DNA-EP for adjuvant personalized treatment.

### NCV generated for human cancer models

**Fig. 6:**
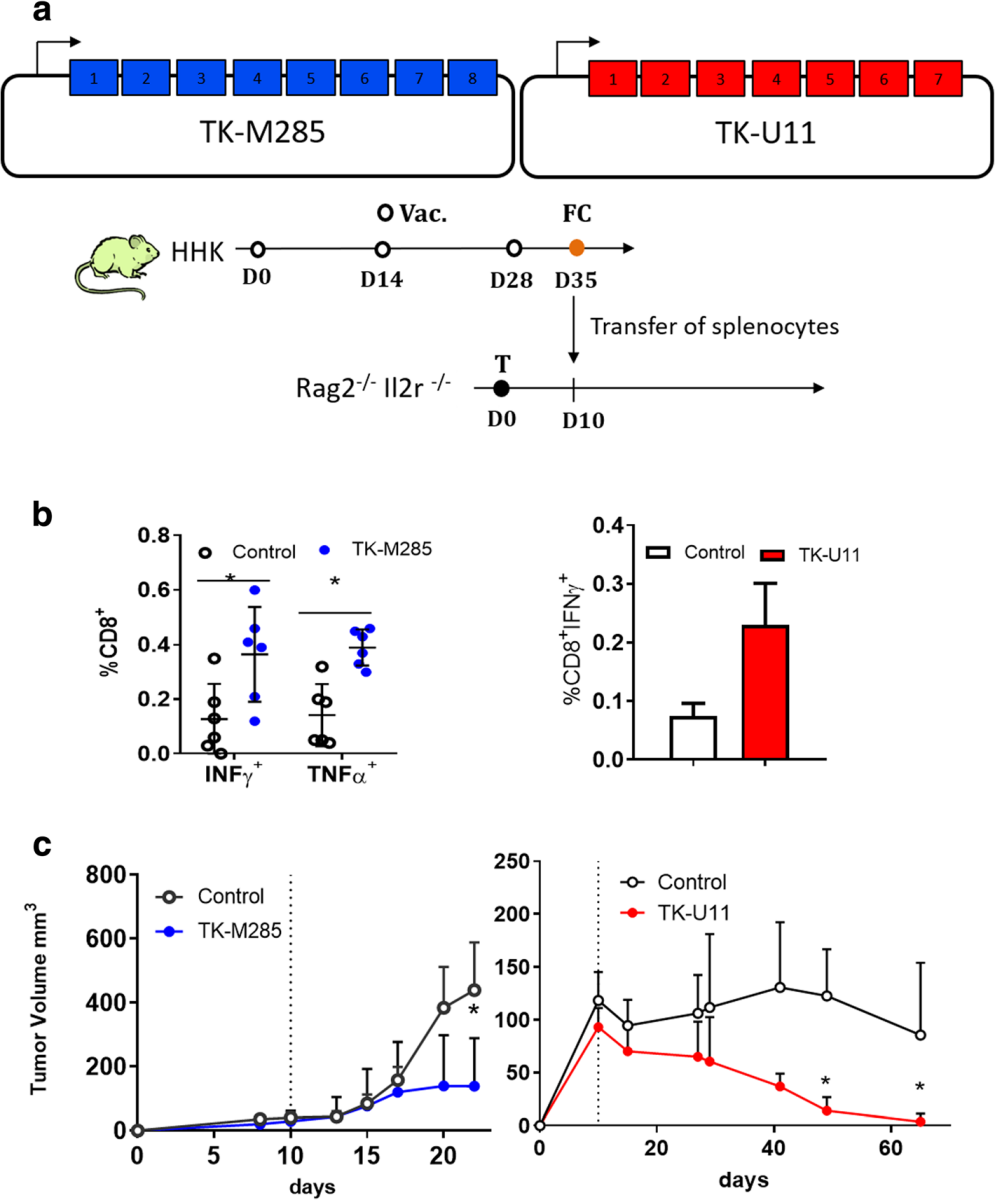
NCV is effective in human derived tumor models. (**a**) Scheme of the vaccine vectors and the vaccination protocol. HHK mice were vaccinated with the TK-M285 (Additional file 1: Table S4) or the TK-U11 (Additional file1: Table S5) vaccine vector and splenocytes transferred in Rag2^−/−^ Il2r^−/−^ tumor bearing mice on day 35. HHK mice were vaccinated either with the TK-M285 or TK-U11 vector at the indicated time points (V) and splenocytes were transferred on day 35 into Rag2^−/−^ Il2r^−/−^ mice bearing tumors (T) of M285 or U11, respectively. (**b**) Immune responses detected in the splenocytes of HHK vaccinated mice at the time of splenocyte transfer. On the left side, a representative experiment of TK-M285 specific immune response of six HHK mice was restimulated with a pool of the eight neoantigen peptides. On the right side, immune responses specific for TK-U11 from four vaccinated mice, bars are SD, paired matched T-test 2-tailed *p < 0.05. (**c**) Tumor growth of M285 and U11 tumor models. Five or six tumor bearing mice were injected i.p. with 5 × 10^6^ splenocytes from HHK vaccinated mice on day 10 (dotted line) and tumor growth followed over time, the data are from one of the two experiments performed. Paired T-test 2-tailed *p < 0.05, bars represent SD

To move closer to the clinical setting and test whether our pipeline was effective in dealing with human tumors, we developed an innovative tumor model based on patient derived tumors and adoptive T cell transfer. Adoptive T cell therapy is effective in melanoma patients and recent evidence suggests that T cells recognize neoantigens [27]. Screening of tumor cells derived from primary tumors for the expression of HLA-A0201 resulted in the selection of the M285 melanoma model [22] and the U11 lung cancer model [21]. As reported for mouse cell lines, neoantigens were selected according to predicted binding to HLA-A0201 and their expression measured by RNAseq (Additional file1: Tables S4 and S5). Selected neoantigens were used to generate the NAM vaccine vectors TK-U11 and TK-M285 (Fig. 6a). We then vaccinated HLA-2.1 transgenic mice (HHK) and transferred splenocytes in Rag2^−/−^ Il2r^−/−^ mice bearing the corresponding human tumors. Neoantigen-specific immune responses were measured in the splenocytes at the time of splenocyte transfer in TK-U11 and TK-M285 vaccinated mice (Fig. 6b). A significant tumor regression was observed in the U11 tumor model while a significant tumor delay was observed in M285 tumor bearing mice (Fig. 6c). These results demonstrate that adoptive transfer of a neoantigen-specific immune response is able to reduce tumor growth of human derived tumors.

## Discussion

In this study, we showed that NCV delivered by DNA-EP are able to provide antitumor effects in murine models and can be used to treat human xenograft tumor models. Our first observation was that well established neoantigens such as M30 [6, 9, 23] did not turn out to be immunogenic when administered through the DNA-EP delivery system. In contrast, the M48 neoantigen was immunogenic using two different NAM vaccine vectors (B1 and B2). Moreover, the two immunogenic neoantigens M21 and M48, which have been previously reported as CD4 epitopes [9], showed a CD8–specificity in our experiments (Additional file 1: Table S2). More importantly, induction of a B16-specific effector T cell response did not correlate with tumor protection. In agreement with this notion, another preclinical study on ovarian cancer showed that immune responses against neoantigens with low affinity did not result in tumor protection [28]. This disappointing result in the context of DNA-EP prompted us to look at the quality of neoantigens in other tumor models.

The analysis of immune responses induced with the twenty predicted neoantigens in MC38 cancer cells and expressed by M1 and M2 vaccines, suggests that DNA-EP induced immunogenicity is driven by high affinity neoantigens. One potential concern regarding neoantigens is safety because of the potential auto-immunity against healthy tissue expressing the cognate self-antigens. In line with previous evidence [14], we showed that the immune response against one of these neoantigens, Reps1, is highly specific compared to the wt epitope. However, further experiments are required to define cross reactivity and potential toxicity. Overall, we used quite a large set of mouse neoantigens (*n* = 31) and reported individual immunological values via the FC analysis. We have to acknowledge that while we confirmed in our vaccination platform the immunogenicity of neoantigens with high affinity such as Reps1 and Adpgk [14], this was not the case for other neoantigens. In contrast, a neoantigen such as Aatf, which is presented on MC38 cells, was not immunogenic when administered as a peptide [14] as well as in our NAM vaccine. Discrepancies with other vaccination methods highlight the fact that neoantigen pipelines, from prediction to delivery methods, need to be experimentally validated. Using the DNA-EP vaccination method, we identified new immunogenic CD8+ neoantigens (Wbp7, Hace1, Tmem135 and Spire1) that had been selected on the basis of predicted high affinity to MHC-I. The minigene DNA-EP technology allowed to accommodate a sufficient number of neoantigens to obtain significant poly-specific immune responses. Our evidence suggests that the quality, as well as the number of neoantigens, are key parameters for a productive immune response.

We observed a strong poly-functional immune response particularly with the M2 vaccine. The Adpgk neoantigen from MC38 cells showed an immune response dominated by IFN-γ upon delivery by a very efficient system based on peptide embedded in liposome disk [23]. In contrast, we observed a clear poly-functional response mostly due to CD8^+^IFN-γ^+^TNF-α^+^ T cells. However, further experiments with more neoantigens compared side by side using different vaccine platforms are required before drawing any conclusions. Interestingly, poly-functionality was observed also in a clinical study, where a personalized vaccine delivered as an RNA for melanoma patients showed poly-functional CD8^+^IFN-γ^+^TNF-α^+^ immune responses [5]. Poly-functionality was not limited to a vaccine-induced immune response but was also reported for natural immune responses against neoantigens in ovarian cancer patients [29].

Our preliminary data with the humanized “immunoavatar” model indicates that transferring splenocytes from HHK vaccinated mice blocks the growth of the melanoma M285 xenografted mice and induces tumor regressions in lung cancer U11 transplanted mice. The current model answers the question whether the NCV can induce an immunogenic immune response specific for the patient in the surrogate model of HLA-A0201 transgenic mice and define the potential efficacy as a means of T cell adoptive transfer. Further improvements to this model will be the use of in vitro primed human T cells against neoantigens and their transfer into patient derived xenografts to prove their efficacy.

## Conclusion

Our study suggests that a vaccine endowed with high poly-specificity and linked to poly-functionality is the most efficient in preventing tumor growth. We did not aim to establish a threshold of poly-specificity or a specific combination of neoantigens but rather to show, in a direct comparison, the superiority of a vaccine encoding more neoantigens. This observation supports the concept that a NCV has the potential to broaden the repertoire of immune responses against cancer, a feature that could be particularly relevant in the treatment of tumors with high heterogeneity [30]. In the described setting for MC38 checkpoint inhibition is very effective. The two different treatment strategies employ differing immunologic mechanisms, and as both are comparable in terms of activity it is reasonable to conclude that NCV approach is a potential alternative to currently established therapies. A second significant aspect is the possibility of inducing a long-lasting response to prevent tumor relapse. We observed a significant tumor delay (Fig. 5) after more than one month after the last vaccination when the response was diminishing. In contrast, mice boosted one week in advance (Fig. 6) were completely protected from the tumor challenge, suggesting that in order to maintain high levels of circulating tumor-specific T cells the protocol requires a further boost.

The possibility of extending the NCV approach to tumors other than melanoma is expected based on the high neoantigen load observed for instance in lung cancer [31]. Here, we can show that a vaccine could be designed using RNAseq data and HLA-A0201 binding prediction, although we are confident that improvements in predictive algorithms or the introduction of in vitro binding or functional assays can further increase the identification of immunogenic neoantigens. This is of particular relevance in the context of tumors for which biopsy material is limited but sufficient for NGS approaches [32]. Finally, it is interesting to note that DNA-EP does not induce any neutralizing immune response as it is the case for viral vaccines. Indeed, we demonstrated the feasibility and the clinical efficacy of a repetitive DNA-EP vaccination in a veterinary trial [33]. The relevance of an adjuvant setting for the development of NCV is in line with the human clinical trials exploiting this approach [4, 5, 34].

Many clinical trials registered on https://clinicaltrials.gov/ with DNA-EP do not indicate any selection criteria for neoantigens, however it would be interesting to explore the immunological and clinical outcomes as we trust that poly-specificity and poly-functionality of high affinity neoantigens will be highly relevant for the success of this approach.

## Additional files

Additional file 1: **Tables S1.** Primers used for resequencing of B16 neoantigens. **Table S2.** B16 selected neoantigens. **Table S3.** Summary results of RNAseq of in vitro and in vivo MC38 cells. **Table S4.** M285 selected peptides. Binding affinity prediction of M285 neoantigens according to NetMHC-4 and RNAseq values. **Table S5.** U11 selected peptides. Binding affinity prediction of U11 neoantigens according to NetMHC-4 and RNAseq values. (DOCX 116 kb)

Additional file 2: **Figure S1.** Representative gating strategy for the identification of neoantigen specific immune responses. **Figure S2.** Comparison of CEA specific immune responses induced by the M1 vaccine vector vs. the full length CEA protein delivered by DNA-EP. **Figure S3.** Comparison in peripheral blood by IFN-γ ICS analysis for the Reps1 neoantigen and cognate WT peptide. **Figure S4.** M2 specific memory T cells. **Figure S5.** M3 specific T cells. **Figure S6.** Immuno modulators prevent MC38 tumor growth. (PPTX 1226 kb)

## Abbreviations

ACK: Ammonium-Chloride- Potassium
CEA: Carcino-Embryonic Antigen
DAI: Differential agretopic index
EP: Electroporation
FBS: Fetal bovine serum
FC: Flow cytometry
HHK: Immunocompetent mice transgenic for HLA-A0201
ICI: Immune checkpoint inhibitors
RPM: Reads per milion
WT: Wild-type

## References

[1] Hu Z, Ott PA, Wu CJ. Towards personalized, tumour-specific, therapeutic vaccines for cancer. Nat. Publ. Gr. [Internet]. Nature Publishing Group; 2017; Available from: 10.1038/nri.2017.131 10.1038/nri.2017.131 PMC6508552 29226910

[2] Aurisicchio L, Pallocca M, Ciliberto G, Palombo F. The perfect personalized cancer therapy: cancer vaccines against neoantigens. J Exp Clin Cancer Res; 2018;37:86. Available from: 10.1186/s13046-018-0751-1 10.1186/s13046-018-0751-1 PMC5910567 29678194

[3] Strønen E, Toebes M, Kelderman S, Van BMM, Yang W, Van RN, et al. Targeting of cancer neoantigens with donor-derived T cell receptor repertoires(1). 2016;2288:1–11. 10.1126/science.aaf2288 27198675

[4] Ott PA, Hu Z, Keskin DB, Shukla SA, Sun J, Bozym DJ, et al. An immunogenic personal neoantigen vaccine for patients with melanoma. Nature [internet]. Nat Publ Group; 2017;547:217–221. Available from: 10.1038/nature22991 10.1038/nature22991 PMC5577644 28678778

[5] Sahin U, Derhovanessian E, Miller M, Kloke BP, Simon P, Löwer M (2017). Personalized RNA mutanome vaccines mobilize poly-specific therapeutic immunity against cancer. Nature [internet]. Nat Publ Group.

[6] Castle JC, Kreiter S, Diekmann J, Löwer M, Van De Roemer N, De Graaf J (2012). Exploiting the mutanome for tumor vaccination. Cancer Res.

[7] Aurisicchio L, Fridman A, Bagchi A, Scarselli E, La Monica N, Ciliberto G. A novel minigene scaffold for therapeutic cancer vaccines. Oncoimmunology [Internet]. 2014;3:e27529. Available from: http://www.tandfonline.com/doi/full/10.4161/onci.27529 10.4161/onci.27529 PMC4002591 24790791

[8] Pierini S, Perales-Linares R, Uribe-Herranz M, Pol JG, Zitvogel L, Kroemer G, et al. Trial watch: DNA-based vaccines for oncological indications. Oncoimmunology. Taylor & Francis; 2017;6:1–17. Available from: 10.1080/2162402X.2017.1398878. 10.1080/2162402X.2017.1398878 PMC5706602 29209575

[9] Kreiter S, Vormehr M, van de Roemer N, Diken M, Löwer M, Diekmann J, et al. Mutant MHC class II epitopes drive therapeutic immune responses to cancer. Nature [Internet]. 2015;520:692–6. Available from: https://www.nature.com/articles/nature14426. 10.1038/nature14426 PMC4838069 25901682

[10] Kranz LM, Diken M, Haas H, Kreiter S, Loquai C, Reuter KC (2016). Systemic RNA delivery to dendritic cells exploits antiviral defence for cancer immunotherapy. Nature.

[11] Duan F, Duitama J, Al Seesi S, Ayres CM, Corcelli SA, Pawashe AP, et al. Genomic and bioinformatic profiling of mutational neoepitopes reveals new rules to predict anticancer immunogenicity. J Exp Med. 2014;211:2231–48. Available from: https://www.ncbi.nlm.nih.gov/pmc/articles/PMC4203949/. 10.1084/jem.20141308 PMC4203949 25245761

[12] Snyder A, Makarov V, Merghoub T, Yuan J, Zaretsky JM, Desrichard A, et al. Genetic basis for clinical response to CTLA-4 blockade in melanoma. N Engl J Med. 2014;2189–99. Available from: https://www.ncbi.nlm.nih.gov/pmc/articles/PMC4315319/. 10.1056/NEJMoa1406498 PMC4315319 25409260

[13] Balachandran VP, Luksza M, Zhao JN, Makarov V, Moral JA, Remark R (2017). Identification of unique neoantigen qualities in long-term survivors of pancreatic cancer. Nature England.

[14] Yadav M, Jhunjhunwala S, Phung QT, Lupardus P, Tanguay J, Bumbaca S, et al. Predicting immunogenic tumour mutations by combining mass spectrometry and exome sequencing. Nature [Internet]. 2014;515:572–6. Available from: http://www.nature.com/doifinder/10.1038/nature14001%5C. 10.1038/nature14001 25428506

[15] Folgiero V, Sorino C, Pallocca M, De Nicola F, Goeman F, Bertaina V, et al. Che-1 is targeted by c-Myc to sustain proliferation in pre-B-cell acute lymphoblastic leukemia. EMBO Rep England. 2018;19. 10.15252/embr.201744871 PMC5835840 29367285

[16] Di Marco M, Astolfi A, Grassi E, Vecchiarelli S, Macchini M, Indio V (2015). Characterization of pancreatic ductal adenocarcinoma using whole transcriptome sequencing and copy number analysis by single-nucleotide polymorphism array. Mol Med Rep.

[17] Garrison E. Marth G. Haplotype-based variant detection from short-read sequencing. 2012:1–9 Available from: http://arxiv.org/abs/1207.3907.

[18] Nielsen M, Lundegaard C, Worning P, Lauemoller SL, Lamberth K, Buus S (2003). Reliable prediction of T-cell epitopes using neural networks with novel sequence representations. Protein Sci United States.

[19] Mennuni C, Calvaruso F, Facciabene A, Aurisicchio L, Storto M, Scarselli E (2005). Efficient induction of T-cell responses to carcinoembryonic antigen by a heterologous prime-boost regimen using DNA and adenovirus vectors carrying a codon usage optimized cDNA. Int J Cancer.

[20] Elia L, Mennuni C, Storto M, Podda S, Calvaruso F, Salucci V (2006). Genetic vaccines against ep-CAM break tolerance to self in a limited subset of subjects: initial identification of predictive biomarkers. Eur J Immunol.

[21] Roscilli G, De Vitis C, Ferrara FF, Noto A, Cherubini E, Ricci A (2016). Human lung adenocarcinoma cell cultures derived from malignant pleural effusions as model system to predict patients chemosensitivity. J Transl Med.

[22] von Euw E, Atefi M, Attar N, Chu C, Zachariah S, Burgess BL (2012). Antitumor effects of the investigational selective MEK inhibitor TAK733 against cutaneous and uveal melanoma cell lines. Mol Cancer.

[23] Kuai R, Ochyl LJ, Bahjat KS, Schwendeman A, Moon JJ (2017). Designer vaccine nanodiscs for personalized cancer immunotherapy. Nat Mater England.

[24] Teku GN, Vihinen M (2018). Pan-cancer analysis of neoepitopes. Sci Rep Springer US.

[25] Eggermont AMM, Blank CU, Mandala M, Long G V, Atkinson V, Dalle S, et al. Adjuvant Pembrolizumab versus Placebo in Resected Stage III Melanoma. N Engl J Med. 2018;NEJMoa1802357. Available from: http://www.nejm.org/doi/10.1056/NEJMoa1802357 10.1056/NEJMoa1802357 29658430

[26] Eggermont AMM, Chiarion-Sileni V, Grob JJ, Dummer R, Wolchok JD, Schmidt H (2015). Adjuvant ipilimumab versus placebo after complete resection of high-risk stage III melanoma (EORTC 18071): a randomised, double-blind, phase 3 trial. Lancet Oncol.

[27] Tran E, Robbins PF, Rosenberg SA (2017). Final common pathway’ of human cancer immunotherapy: targeting random somatic mutations. Nat Immunol.

[28] Martin SD, Brown SD, Wick DA, Nielsen JS, Kroeger R. Twumasi-boateng K, et al. Low Mutation Burden in Ovarian Cancer May Limit the Utility of Neoantigen-Targeted Vaccines. 2016:1–22. Available from:. 10.1371/journal.pone.0155189. 10.1371/journal.pone.0155189 PMC4871527 27192170

[29] Bobisse S, Genolet R, Roberti A, Tanyi JL, Racle J, Stevenson BJ, et al. Sensitive and frequent identification of high avidity neo-epitope specific CD8+T cells in immunotherapy-naive ovarian cancer. Nat Commun [internet]. Springer US; 2018;9:1–10. Available from: 10.1038/s41467-018-03301-0 10.1038/s41467-018-03301-0 PMC5854609 29545564

[30] Mcgranahan N, Furness AJS, Rosenthal R, Ramskov S, Lyngaa R, Saini SK, et al. Clonal neoantigens elicit T cell immunoreactivity and sensitivity to immune checkpoint blockade. Science (80-. ). [Internet]. 2016;351:1463–9. Available from: http://www.sciencemag.org/cgi/doi/10.1126/science.aaf1490 10.1126/science.aaf1490 PMC4984254 26940869

[31] Yarchoan M, Johnson BA 3rd, Lutz ER, Laheru DA, Jaffee EM. Targeting neoantigens to augment antitumour immunity. Nat Rev Cancer England; 2017;17:209–222. 10.1038/nrc.2016.154 PMC5575801 28233802

[32] Bassani-Sternberg M, Bräunlein E, Klar R, Engleitner T, Sinitcyn P, Audehm S, et al. Direct identification of clinically relevant neoepitopes presented on native human melanoma tissue by mass spectrometry. Nat. Commun. [Internet]. 2016;7:13404. Available from: http://www.nature.com/doifinder/10.1038/ncomms13404 10.1038/ncomms13404 PMC5121339 27869121

[33] Peruzzi D, Gavazza A, Mesiti G, Lubas G, Scarselli E, Conforti A, et al. A vaccine targeting telomerase enhances survival of dogs affected by B-cell lymphoma. Mol. Ther. [internet]. Nat Publ Group; 2010;18:1559–67. Available from: 10.1038/mt.2010.104 10.1038/mt.2010.104 PMC2927056 20531395

[34] Carreno BM, Magrini V, Becker-Hapak M, Kaabinejadian S, Hundal J, Petti AA, et al. Cancer immunotherapy. A dendritic cell vaccine increases the breadth and diversity of melanoma neoantigen-specific T cells. Science (80-. ). [internet]. 2015;348:803–8. Available from: http://science.sciencemag.org/content/348/6236/803.long. 10.1126/science.aaa3828 PMC4549796 25837513

