# Supplementary material for: Poly-specific neoantigen-targeted cancer vaccines delay patient derived tumor growth
Source: J Exp Clin Cancer Res. 2019 Feb 14;38:78. doi: 10.1186/s13046-019-1084-4 (PMC6376688; doi:10.1186/s13046-019-1084-4)
Supplement: Supplementary file 1 — Tables S1. Primers used for resequencing of B16 neoantigens. Table S2. B16 selected neoantigens. Table S3. Summary results of RNAseq of in vitro and in vivo MC38 cells. Table S4. M285 selected peptides. Binding affinity prediction of M285 neoantigens according to NetMHC-4 and RNAseq values. Table S5. U11 selected peptides. Binding affinity prediction of U11 neoantigens according to NetMHC-4 and RNAseq values. (DOCX 116 kb) [file 13046_2019_1084_MOESM1_ESM.docx]

Table S1

Primers used for resequencing of B16 neoantigens

| Gene | 5' | 3' | ID |
| --- | --- | --- | --- |
| Def8 | GCCAATACTACTGCAGCCAC | CTCCAAGCCTCCACTCCACC | chr8_125980397 |
| Tubb3 | CCGTGGGCTCAAAATGTCAT | TACTGCTGGTACTCGGACAC | chr8_125945433 |
| Tm9sf3 | GCAGGCCTTGTATTAGAATGGT | GGGCATTCCTTATCCCAGCT | chr19_41306867 |
| Sema3b | AACTGCAGAAAGTCCCGGTA | CTCTGGCGTGTATCTTTGCG | chr9_107501364 |
| Ppp1r7 | TCTAGCTAAGATTCCCACTCCT | CCAGCTCCAACATTTGCAGT | chr1_95249164 |
| Obsl1 | ACTGTCCTGGCCTGCTTGGC | GGCCACGTGTGTTGGATGCG | chr1_75483330 |
| Mthfd1l | AAGCAAGGTCCGTCATCTGA | CATTGTGGTCTTAGGCTCACC | chr10_6338696 |
| Mkrn1 | CCGGCTGCTCATACCTCAT | GCATTGCAGCCTGTTCATTG | chr6_39350454 |
| Kif18b | CTCCAAGAGTCAGGCTACCT | GGACCTCAACACCACCTTCA | chr11_102769470 |
| Dag1 | CCGAGTTGTGGTAGTTGACG | CACGGTTCCTGGCCAGATTC | chr9_108110998 |
| Cpsf3l | AGCTCTTCATCACCTGGACC | AGACCTTTGATCAGCAGCCT | chr4_155261078 |
| Atp11a | GCTTTGCCTTCTCTGTGTCC | TGTCGTTCTCCCTGTTCAGT | chr8_12834636 |

**Table S2**

B16 selected neoantigens. Neoantigens from B16 cells are expressed by the vaccine vectors B1 and B2 (Fig. 1). Neoantigens were predicted as 9 mer CD8 peptide by NetMHC4 and are indicated in italic for the Db haplotype and are underlined for he Kb haplotype. Predicted binding values are expressed as affinity nM, mutated residues are in bold. Immune responses against individual neoantigens were analyzed by FC as described in M&M

| Mutation | Mutated sequence utilized | *Db* | Kb | CD8 | CD4 |
| --- | --- | --- | --- | --- | --- |
| M27 | REGVE*LCPGNKYE****M***RRHGTTHSLVIHD | 4375 | 19715 | - | - |
| M48 | SHCHWNDLAVIPA***G****VVHNWDFE*PRKVS | 15391 | 10685 | + | - |
| M44 | EFKHIKAFDR*TFA****N****NPGPM*VVFATPGM | 8962 | 9770 | - | - |
| M29 | IPSGTT*ILNCFHD****V****L*SGKLSGGSPGVP | 12226 | 944 | - | - |
| M20 | FRRKAFLHW*YTGE****A****MDEM*EFTEAESNM | 8060 | 5573 | - | - |
| M21 | SSPDEV*ALVEGVQ****S****L*GFTYLRLKDNYM | 23975 | 492 | + | - |
| M28 | NIEGIDKL*TQLKK****P****FLV*NNKINKIENI | 21431 | 3183 | - | - |
| M24 | TAVITPP*TTTTKK****A****RV*STPKPATPSTD | 44378 | 3584 | - | - |
| M36 | CGTAFFINF*IAIY****H****HASR*AIPFGTMVA | 38680 | 702 | - | - |
| M45 | ECRITSN*FVIPSE****Y****WV*EEKEEKQKLIQ | 7030 | 7240 | - | - |
| M30 | PSKPSF*QEFVDWE****N****V*SPELNSTDQPFL | 35041 | 12461 | - | - |

Table S3

Summary results of RNAseq of *in vitro* and *in vivo* MC38 cells

|  | MC38 in vitro | | | | | MC38 in vivo | | | | |  |  |  |  |  |  |  |
| --- | --- | --- | --- | --- | --- | --- | --- | --- | --- | --- | --- | --- | --- | --- | --- | --- | --- |
| **Gene** | **RPM** | **total** | **mutated** | **ref.** | **RNAseq Allele freq.** | **RPM** | **total** | **mutated** | **ref.** | **RNAseq Allele freq.** | **Peptide WT** | **Peptide Mut** | **seqnames** | **start** | **end** | **ref** | **alt** |
| Wbp7 | 0,51 | 17 | 15 | 2 | 88,2% | 0,29 | 15 | 10 | 5 | 66,7% | SNFHFMCAR | SNFHFMCAL | 7 | 31362389 | 31362389 | C | A |
| Hace1 | 0,20 | 26 | 6 | 20 | 23,1% | 0,23 | 30 | 8 | 22 | 26,7% | QINAFLQGF | QIYAFLQGF | 10 | 45406468 | 45406468 | A | T |
| Hdgfrp2 | 2,84 | 143 | 84 | 59 | 58,7% | 1,37 | 79 | 47 | 32 | 59,5% | KGYPHWPAR | KGYPHWPAL | 17 | 56219248 | 56219248 | G | T |
| Kpna6 | 1,99 | 245 | 59 | 186 | 24,1% | 1,45 | 232 | 50 | 182 | 21,6% | CTLQFEAAW | CTLQFEAAL | 4 | 129332792 | 129332792 | C | A |
| Aurkaip1 | 2,94 | 273 | 87 | 186 | 31,9% | 2,18 | 276 | 75 | 201 | 27,2% | RTRFLRRKV | RTRFLRLKV | 4 | 155206872 | 155206872 | G | T |
| Srebf2 | 0,41 | 23 | 12 | 11 | 52,2% | 0,26 | 17 | 9 | 8 | 52,9% | HSFVDSVGF | HSFVYSVGF | 15 | 82025394 | 82025394 | G | T |
| Mttp | 0,24 | 23 | 7 | 16 | 30,4% | 0,12 | 15 | 4 | 11 | 26,7% | TGYVERSPR | TGYVERSPL | 3 | 137767743 | 137767743 | C | A |
| Nle1 | 0,41 | 23 | 12 | 11 | 52,2% | 0,35 | 33 | 12 | 21 | 36,4% | MALSTDYAL | MALSTYYAL | 11 | 82717814 | 82717814 | C | A |
| Zbtb24 | 0,24 | 11 | 7 | 4 | 63,6% | 0,26 | 17 | 9 | 8 | 52,9% | SLLEHMSLH | SLLEHMSLL | 10 | 41174996 | 41174996 | A | T |
| Hnrnpf | 29,62 | 1232 | 877 | 355 | 71,2% | 22,52 | 1186 | 775 | 411 | 65,3% | GYVVKLRGL | SYVVKLRGL | 6 | 117873802 | 117873802 | G | A |
| Dpagt1 | 1,15 | 59 | 34 | 25 | 57,6% | 1,19 | 69 | 41 | 28 | 59,4% | ASIIVFNLV | ASIIVFNLL | 9 | 44137208 | 44137208 | G | T |
| Tmem135 | 0,88 | 84 | 26 | 58 | 31,0% | 0,44 | 64 | 15 | 49 | 23,4% | FALMNRKAL | FALMNLKAL | 7 | 96290463 | 96290463 | C | A |
| Aatf | 3,14 | 143 | 93 | 50 | 65,0% | 1,77 | 106 | 61 | 45 | 57,5% | MAPIDHTAM | MAPIDHTTM | 11 | 84256087 | 84256087 | C | T |
| Spire1 | 3,51 | 109 | 104 | 5 | 95,4% | 2,27 | 84 | 78 | 6 | 92,9% | SAIRSYQDV | SAIRSYQYV | 18 | 67712239 | 67712239 | C | A |
| Zbtb40 | 0,71 | 28 | 21 | 7 | 75,0% | 0,61 | 25 | 21 | 4 | 84,0% | KSFHFYCRL | KSFHFYCPL | 4 | 136551378 | 136551378 | C | G |
| Slc12a4 | 2,06 | 83 | 61 | 22 | 73,5% | 1,08 | 56 | 37 | 19 | 66,1% | LSAARYALL | LSASRYALL | 8 | 108471012 | 108471012 | C | A |
| Nfe2l2 | 4,69 | 146 | 139 | 7 | 95,2% | 3,20 | 124 | 110 | 14 | 88,7% | ASYSQVAHI | ASYSLVAHI | 2 | 75517221 | 75517221 | T | A |
| Herc6 | 0,37 | 13 | 11 | 2 | 84,6% | 3,46 | 129 | 119 | 10 | 92,2% | CGYEHTAVL | CVYEHTAVL | 6 | 57550025 | 57550025 | G | T |
| Copb2 | 4,66 | 198 | 138 | 60 | 69,7% | 5,00 | 269 | 172 | 97 | 63,9% | MSYFLQGKL | MSYFLQGTL | 9 | 98486565 | 98486565 | A | C |
| Reps1 | 0,91 | 60 | 27 | 33 | 45,0% | 0,61 | 48 | 21 | 27 | 43,8% | AQLPNDVVL | AQLANDVVL | 10 | 17775901 | 17775901 | C | G |
| Adpgk | 0,61 | 50 | 18 | 32 | 36,0% | 0,32 | 44 | 11 | 33 | 25,0% | ASMTNRELM | ASMTNMELM | 9 | 59161630 | 59161630 | G | T |

Table S4

**M285 selected peptides**. Binding affinity prediction of M285 neoantigens according to NetMHC-4 and RNAseq values. Neoantigens from M285 cells are expressed by vaccine vector TK-M285 depicted in Fig. 7. Mutated residues are in bold, neoantigens predicted as 9 mer CD8 peptide by NetMHC4 are underlined

Table S5

**U11 selected peptides**. Binding affinity prediction of U11 neoantigens according to NetMHC-4 and RNAseq values. Neoantigens from U11 cells are expressed by vaccine vector TK-U11 depicted in Fig. 7. Mutated residues are in bold, neoantigens predicted as 9 mer CD8 peptide by NetMHC4 are underlined.

| **Neoant. #** | Peptide | HLA-A0201 nM | peptide_string | **wt_peptide_string** | Chr | Start | ref Allele | alt Allele | ref RPM | alt RPM | ref Count | alt Count | total Count | RNAseq Allele freq. | Gene |
| --- | --- | --- | --- | --- | --- | --- | --- | --- | --- | --- | --- | --- | --- | --- | --- |
| 1 | NTMEDVVLV | 24.1 | FTDGITNKLIGCY**M**GNTMEDVVLVRIYG | FTDGITNKLIGCYVGNTMEDVVLVRIYG | chr12 | 22796739 | G | A | 1,33 | 0,90 | 61 | 41 | 102 | 40,2% | ETNK1 |
| 2 | GMWQEKVTV | 36.2 | RVMVLNRSGMWQE**K**VTVPSVQTFLIPEA | RVMVLNRSGMWQEEVTVPSVQTFLIPEA | chr17 | 41170768 | C | T | 1,79 | 1,55 | 82 | 71 | 153 | 46,4% | VAT1 |
| 3 | SLYNNKCLV | 40.1 | SPSVCSNLAAKHS**L**YNNKCLVHIVRSTS | SPSVCSNLAAKHSFYNNKCLVHIVRSTS | chr22 | 41744160 | C | G | 3,69 | 1,49 | 169 | 68 | 237 | 28,7% | ZC3H7B |
| 4 | FLIRYAKTL | 49.0 | SLRKEVCQLLPFL**I**RYAKTLYEEAEEAN | SLRKEVCQLLPFLVRYAKTLYEEAEEAN | chr1 | 36028113 | G | A | 1,88 | 0,74 | 86 | 34 | 120 | 28,3% | NCDN |
| 5 | YMGNTMEDV | 51.2 | FTDGITNKLIGCY**M**GNTMEDVVLVRIYG | FTDGITNKLIGCYVGNTMEDVVLVRIYG | chr12 | 22796739 | G | A | 1,33 | 0,90 | 61 | 41 | 102 | 40,2% | ETNK1 |
| 6 | HLTSVTLEL | 60.8 | GKIIMTAAAKHLT**S**VTLELGGKSPCYVD | GKIIMTAAAKHLTPVTLELGGKSPCYVD | chr17 | 19645390 | G | A | 2,05 | 0,92 | 94 | 42 | 136 | 30,9% | ALDH3A1 |
| 7 | AIYALLTYM | 68.6 | SGDMGLGVPFNIA**I**YALLTYMIAHITGL | SGDMGLGVPFNIASYALLTYMIAHITGL | chr18 | 670821 | G | T | 6,43 | 3,67 | 294 | 168 | 462 | 36,4% | TYMS |
