# Supplementary material for: Poly-specific neoantigen-targeted cancer vaccines delay patient derived tumor growth
Source: J Exp Clin Cancer Res. 2019 Feb 14;38:78. doi: 10.1186/s13046-019-1084-4 (PMC6376688; doi:10.1186/s13046-019-1084-4)
Supplement: Supplementary file 2 — Figure S1. Representative gating strategy for the identification of neoantigen specific immune responses. Figure S2. Comparison of CEA specific immune responses induced by the M1 vaccine vector vs. the full length CEA protein delivered by DNA-EP. Figure S3. Comparison in peripheral blood by IFN-γ ICS analysis for the Reps1 neoantigen and cognate WT peptide. Figure S4. M2 specific memory T cells. Figure S5. M3 specific T cells. Figure S6. Immuno modulators prevent MC38 tumor growth. (PPTX 1226 kb) [file 13046_2019_1084_MOESM2_ESM.pptx]

## Slide 1
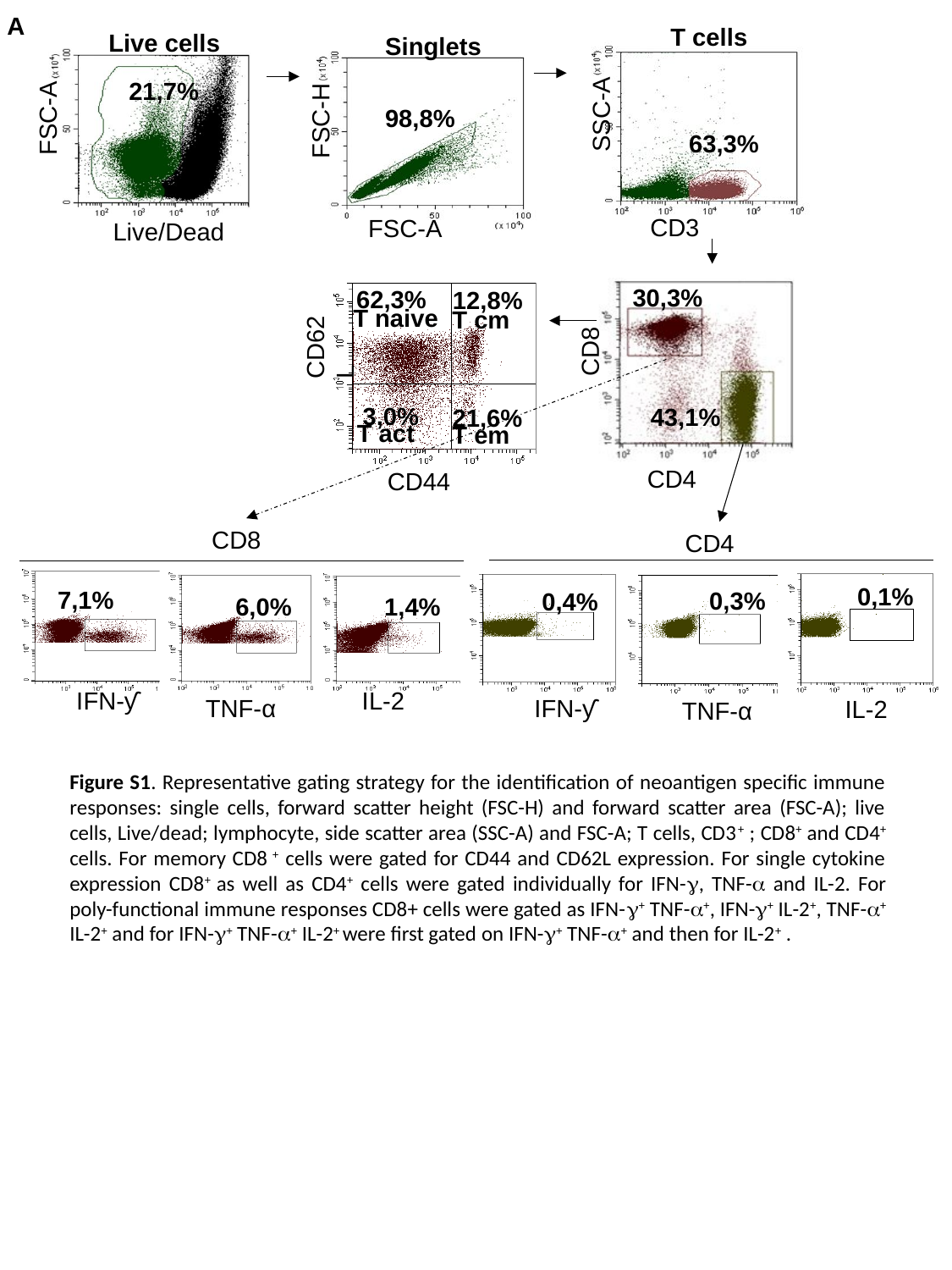

A
T cells
SSC-A
63,3%
CD3
Live cells
21,7%
FSC-A
Live/Dead
Singlets
98,8%
FSC-H
FSC-A
B
30,3%
CD8
43,1%
CD4
62,3%
12,8%
CD62L
3,0%
21,6%
CD44
T naive
T cm
T act
T em
CD8
CD4
0,3%
0,4%
IFN-ƴ
IL-2
TNF-α
7,1%
CD8
IFN-ƴ
6,0%
TNF-α
1,4%
IL-2
0,1%
Figure S1. Representative gating strategy for the identification of neoantigen specific immune responses: single cells, forward scatter height (FSC-H) and forward scatter area (FSC-A); live cells, Live/dead; lymphocyte, side scatter area (SSC-A) and FSC-A; T cells, CD3+ ; CD8+ and CD4+ cells. For memory CD8 + cells were gated for CD44 and CD62L expression. For single cytokine expression CD8+ as well as CD4+ cells were gated individually for IFN-g, TNF-a and IL-2. For poly-functional immune responses CD8+ cells were gated as IFN-g+ TNF-a+, IFN-g+ IL-2+, TNF-a+ IL-2+ and for IFN-g+ TNF-a+ IL-2+ were first gated on IFN-g+ TNF-a+ and then for IL-2+ .

## Slide 2
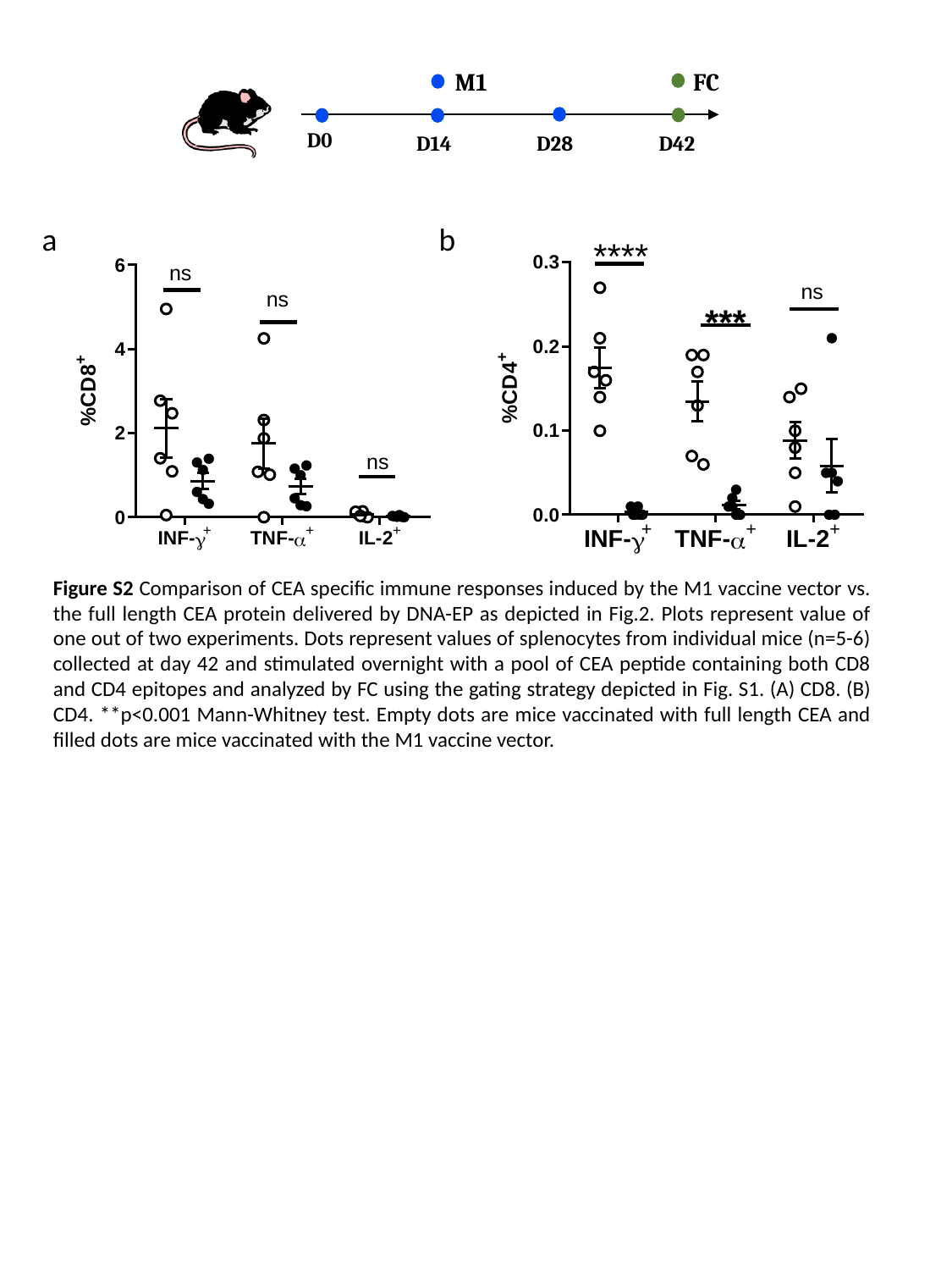

M1
FC
D0
D14
D42
D28
a
b
Figure S2 Comparison of CEA specific immune responses induced by the M1 vaccine vector vs. the full length CEA protein delivered by DNA-EP as depicted in Fig.2. Plots represent value of one out of two experiments. Dots represent values of splenocytes from individual mice (n=5-6) collected at day 42 and stimulated overnight with a pool of CEA peptide containing both CD8 and CD4 epitopes and analyzed by FC using the gating strategy depicted in Fig. S1. (A) CD8. (B) CD4. **p<0.001 Mann-Whitney test. Empty dots are mice vaccinated with full length CEA and filled dots are mice vaccinated with the M1 vaccine vector.

## Slide 3
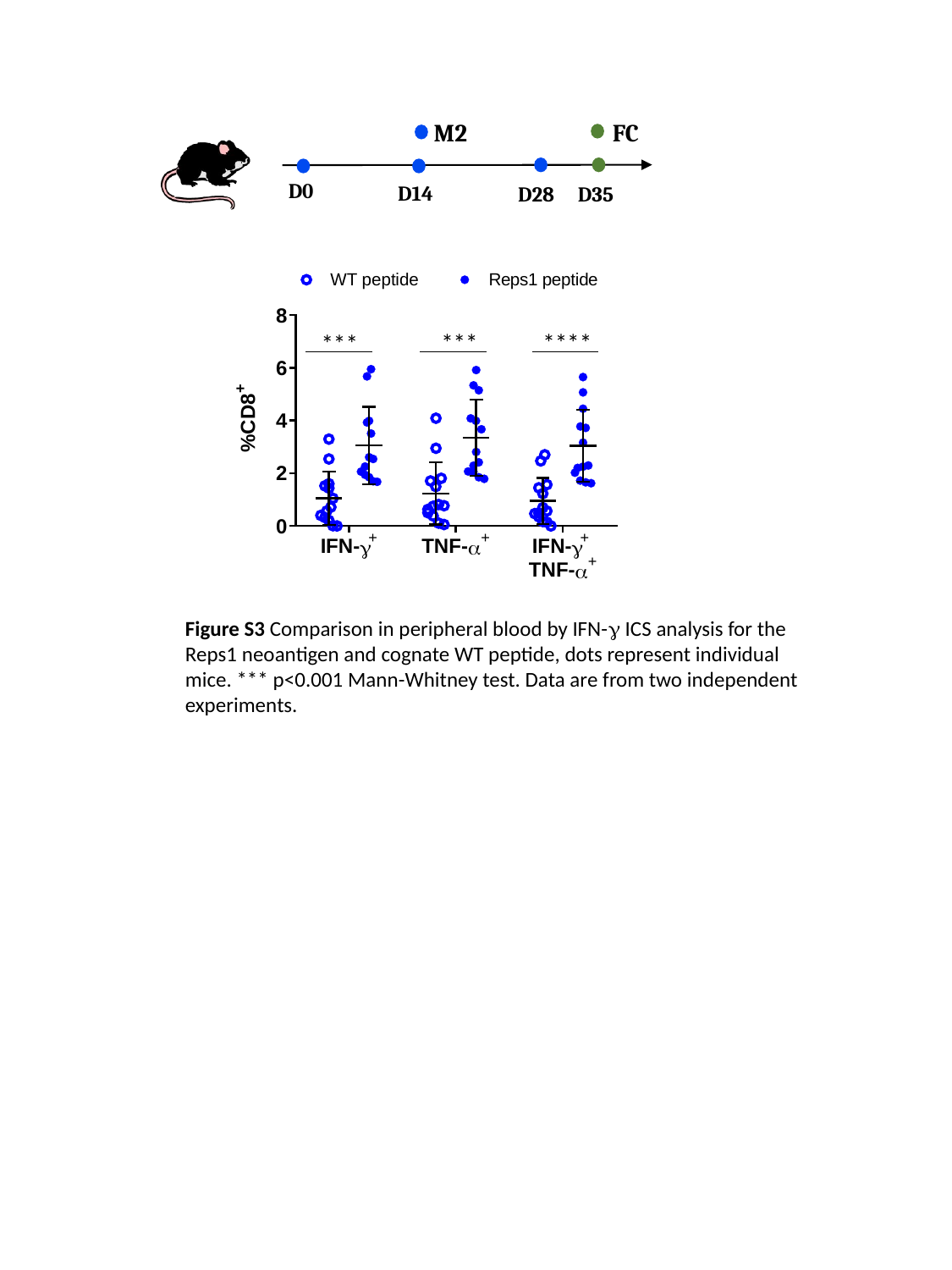

M2
FC
D0
D14
D28
D35
****
***
***
Figure S3 Comparison in peripheral blood by IFN-g ICS analysis for the Reps1 neoantigen and cognate WT peptide, dots represent individual mice. *** p<0.001 Mann-Whitney test. Data are from two independent experiments.

## Slide 4
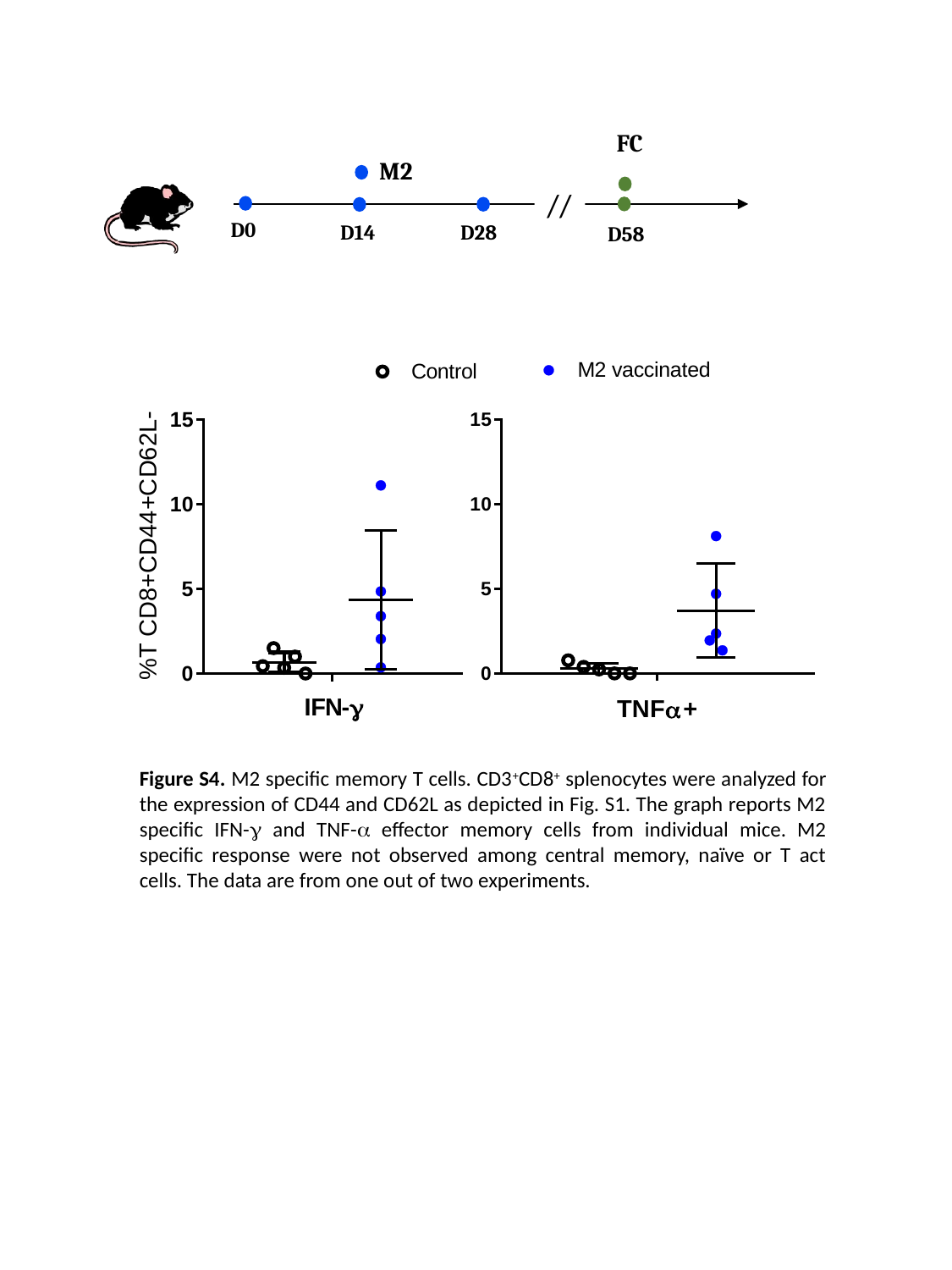

FC
M2
//
D0
D14
D28
D58
Figure S4. M2 specific memory T cells. CD3+CD8+ splenocytes were analyzed for the expression of CD44 and CD62L as depicted in Fig. S1. The graph reports M2 specific IFN-g and TNF-a effector memory cells from individual mice. M2 specific response were not observed among central memory, naïve or T act cells. The data are from one out of two experiments.

## Slide 5
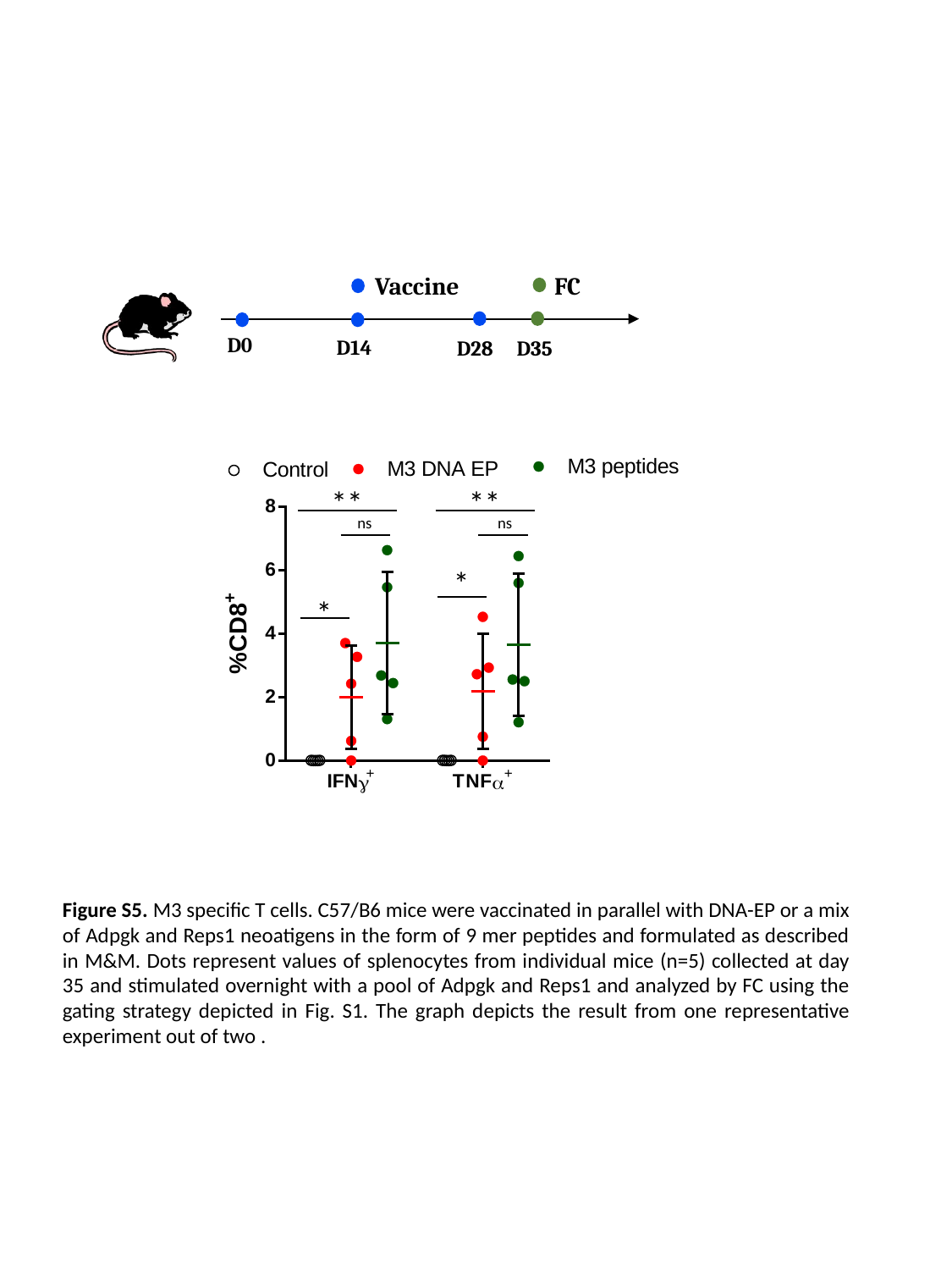

Vaccine
FC
D0
D14
D28
D35
**
**
ns
ns
*
*
Figure S5. M3 specific T cells. C57/B6 mice were vaccinated in parallel with DNA-EP or a mix of Adpgk and Reps1 neoatigens in the form of 9 mer peptides and formulated as described in M&M. Dots represent values of splenocytes from individual mice (n=5) collected at day 35 and stimulated overnight with a pool of Adpgk and Reps1 and analyzed by FC using the gating strategy depicted in Fig. S1. The graph depicts the result from one representative experiment out of two .

## Slide 6
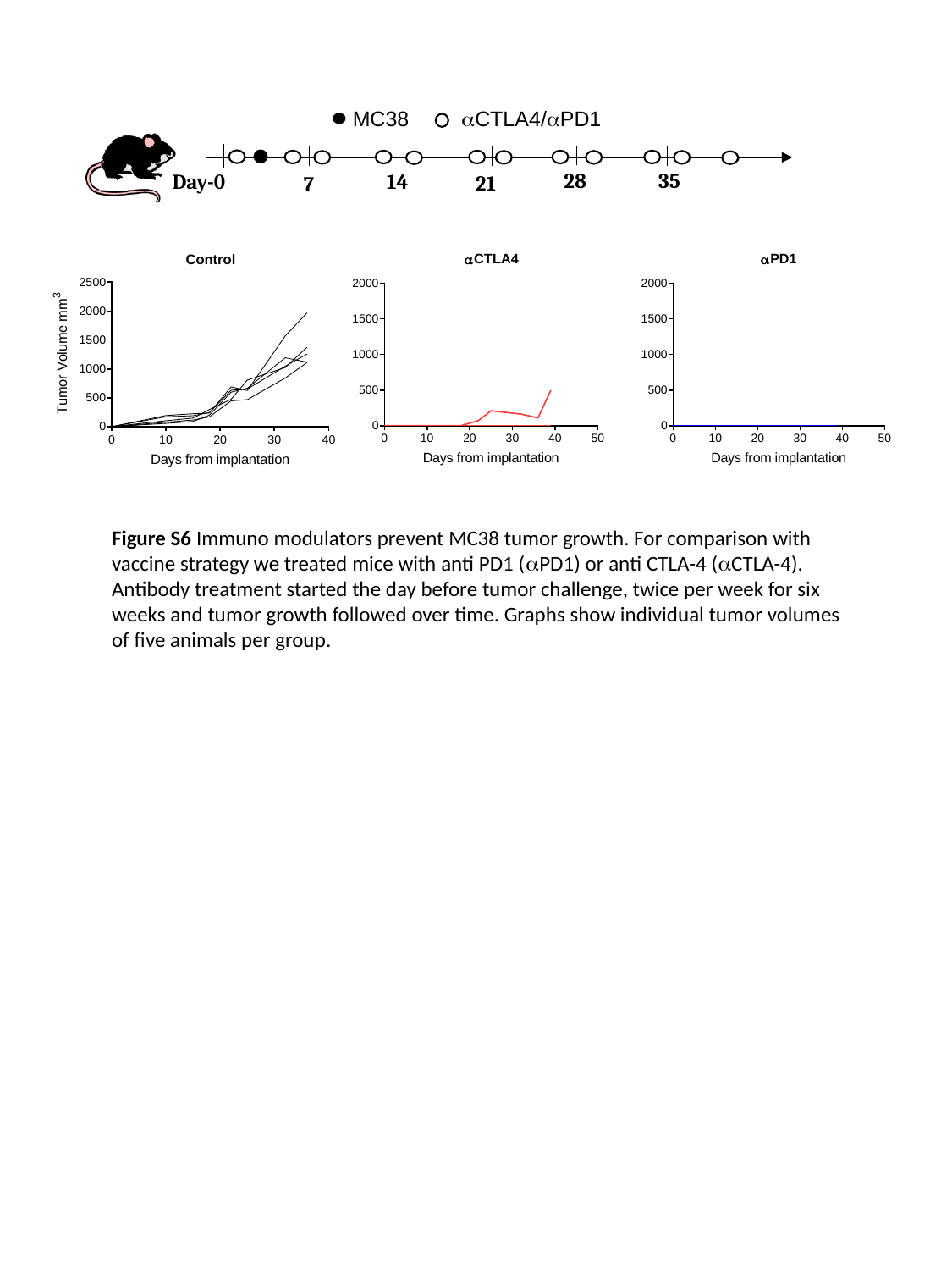

aCTLA4/aPD1
MC38
 35
 28
Day-0
 14
 21
 7
Figure S6 Immuno modulators prevent MC38 tumor growth. For comparison with vaccine strategy we treated mice with anti PD1 (aPD1) or anti CTLA-4 (aCTLA-4). Antibody treatment started the day before tumor challenge, twice per week for six weeks and tumor growth followed over time. Graphs show individual tumor volumes of five animals per group.
